# Reassessment of chitosanase substrate specificities and classification

**DOI:** 10.1038/s41467-017-01667-1

**Published:** 2017-11-22

**Authors:** Tobias Weikert, Anna Niehues, Stefan Cord-Landwehr, Margareta J. Hellmann, Bruno M. Moerschbacher

**Affiliations:** Institute for Biology and Biotechnology of Plants, University of Münster, Schlossplatz 8, 48143 Münster, Germany

## Abstract

Chitosanases can be used to produce partially acetylated chitosan oligosaccharides (paCOS) for different applications, provided they are thoroughly characterized. However, recent studies indicate that the established classification system for chitosanases is too simplistic. Here, we apply a highly sensitive method for quantitatively sequencing paCOS to reassess the substrate specificities of the best-characterized class I–III chitosanases. The enzymes’ abilities to cleave bonds at GlcNAc residues positioned at subsite (−1) or (+1), on which the classification system is based, vary especially when the substrates have different fractions of acetylation (F_*A*_). Conflicts with the recent classification are observed at higher F_*A*_, which were not investigated in prior specificity determinations. Initial analyses of pectin-degrading enzymes reveal that classifications of other polysaccharide-degrading enzymes should also be critically reassessed. Based on our results, we tentatively suggest a chitosanase classification system which is based on specificities and preferences of subsites (−2) to (+2).

## Abstract (web summary)

Chitosanases are classified according to their specificity in cleaving bonds at GlcNAc residues but the current system may be too simplistic. Here, the authors use quantitative mass spectrometry to revisit chitosanase specificity and propose additional determinants for their classification.

## Introduction

Heteropolysaccharides such as glycosaminoglycans, pectins, or chitosans are structurally and functionally important biopolymers whose bioactivities are often conveyed by oligomeric breakdown products upon partial enzymatic degradation. If the degrading enzymes involved possess sequence specificity toward their substrates because their substrate-binding site consists of multiple subsites, each binding one glycosyl residue with defined preferences for different sugar units, these subsite specificities will determine the sequence and, hence, bioactivity of the resulting oligomeric products. Thus, differences in their subsite specificities are often used to classify polysaccharide-degrading enzymes, but analytical tools to accurately determine these specificities are often not available. Here, we describe the use of a recently developed quantitative mass spectrometric sequencing method for the detailed analysis of partially acetylated chitosan oligomers to quantitatively reanalyze the subsite specificities of well-described chitosanases. The results lead us to challenge the current classification of chitosanases into classes I–IV based on their subsite specificities, with potential implications for other polysaccharide-degrading enzymes.

Chitosanases (E.C. 3.2.1.132) can be used to specifically produce partially acetylated chitosan oligosaccharides (paCOS) from polymeric chitosans, which have been extensively studied in the last few decades because they have numerous beneficial bioactivities and are nontoxic, nonallergenic, and biodegradable functional biopolymers^1–7^. Chitosans and their paCOS are derivatives of the structural biopolymer chitin and are composed of β-1,4-linked *N*-acetyl-d-glucosamine (GlcNAc, A) and d-glucosamine (GlcN, D) units^6,8^. As they can differ in their degree of polymerization (DP), fraction of acetylation (F_*A*_), and pattern of acetylation (PA), it is highly challenging to produce defined and reliably bioactive chitosans/paCOS^7^.

Of the chitin-derived products, paCOS have sparked considerable interest because their reduced chain length (lower DP values) results in an increase in solubility in aqueous solutions and can change their bioactivity^9^. However, recent studies indicate that to achieve paCOS with reproducible bioactivities, one must consider not only their DP and F_*A*_, but also their PA, i.e., the sequence of GlcN and GlcNAc units^10^. Therefore, utilizing enzymes like chitosanases, which specifically catalyze the hydrolysis of the β-1,4-glycosidic bonds of GlcN and/or GlcNAc^7,11^, could help to guarantee reliable production of paCOS with desired patterns, at least at their reducing and nonreducing ends, as these mirror the subsite specificities left and right of the cleavage site of the enzyme used, respectively.

**Fig. 1:**
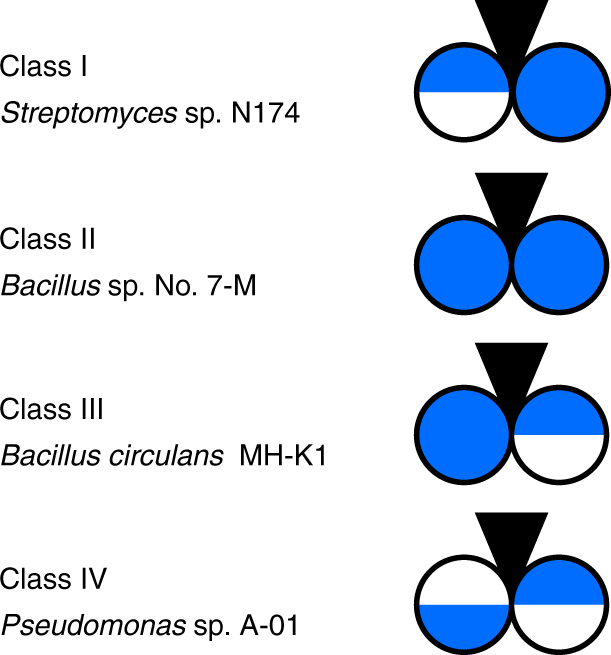
Chitosanase classification according to cleavage specificity with the respective origins for example enzymes. A-units are represented in white, and D-units are represented in blue. For a position, in which either an A-unit or a D-unit can be present, the circle is half-white and half-blue. The point of cleavage is indicated by a black inverted triangle

Chitosanases are grouped into both families and classes: they are grouped into six glycoside hydrolase (GH) families based on their amino acid sequence^12–15^, and they are grouped into four classes based on their cleavage specificity^16,17^ (Fig. 1). All chitosanases can cleave D–D, while class I chitosanases, for example, from *Streptomyces* sp. N174 (belonging to GH 46), can additionally cleave A–D^16–20^; class III chitosanases, like from *Bacillus circulans* MH-K1 (also belonging to GH 46), can, in addition to D–D, also cleave D–A^21–23^. Class II enzymes, such as the *Bacillus* sp. No. 7-M chitosanase (belonging to GH 8) are restricted to bonds between D–D^24,25^; the recently introduced class IV GH 46 chitosanases from *Pseudomonas* sp. A-01^26^ and *Amycolatopsis* sp. CsO-2^27^ can cleave all bonds except A–A^17^. Strikingly, the chitosanase from *Streptomyces coelicolor* A3(2) can cleave all bonds even including A–A, though it prefers D-units^28^; for this type of specificity, no class exists.

While a chitosanase’s class gives initial information about its products’ potential PA, it is the overall efficiency of a chitosanase to cleave different patterns that determines the composition and constitution of the produced paCOS. For example, for a chitosanase from *Streptomyces* sp. N174, previous studies showed that the enzyme can still efficiently cleave chitosans with F_*A*_ in the range of 0.34–0.61^29^, while most chitosanases can only efficiently cleave chitosans in the F_*A*_ range of 0–0.30^11^. Thus, this chitosanase generally seems to act more efficiently than other chitosanases on polymers containing more A-units. More recent studies have indicated that some enzymes cleave different patterns of acetylation with different efficiencies, such that the product composition depends, to some extent, on the substrate’s F_*A*_^20,28^. Furthermore, high substrate concentrations can result in substrate inhibition and reduce the cleavage efficiency^29,30^. Therefore, it might be misleading to compare substrate specificities, and hence use them to classify chitosanases, if these comparisons are done under different conditions (F_*A*_ of the substrate, incubation time, substrate concentration, etc).

To gain more detailed insight into chitosanase substrate specificities, our study focuses on reassessing the substrate specificities of chitosanases from different classes under equal conditions using state-of-the-art techniques^31^. We have analyzed the well-characterized model chitosanases from *Streptomyces* sp. N174 (CSN-174, representing class I), *Bacillus* sp. No. 7-M (CSN-7M, representing class II), and *Bacillus circulans* MH-K1 (CSN-MHKI, representing class III). Additionally, we analyze an unclassified GH 8 chitosanase from *Bacillus* sp. MN (CSN-MN) that we have worked with extensively^32,33^. This chitosanase shows 97% sequence identity to CSN-7M at the amino acid level, suggesting that it belongs to class II, but preliminary analyses suggested that it rather behaves like a class III chitosanase. Therefore, we include this enzyme in our study for comparison. Finally, as the class IV group is rather new^26^, we do not investigate these enzymes in this work. For the enzymes investigated, we use the recently developed method of quantitative sequencing of paCOS^31^ to obtain quantitative data that have been lacking in previous studies. This allows us to not only identify absolute specificities of the subsites (−2) to (+2) for either GlcN or GlcNAc, but to also quantify relative preferences for either of them. We investigate the substrate specificity of well-known chitosanases over a wide range of substrate F_*A*_s at different time points during incubation and compare these preferences to the established chitosanase classification system. We find that the enzymes differ in their subsites’ preferences for GlcN vs. GlcNAc units. Absolute specificities for GlcN are observed in all four enzymes at their (−2) subsite, and in some also at their (−1) subsite, while a more or less strong preference for GlcN over GlcNAc is observed at the (+1) and (+2) subsites of all enzymes.

## Results

### Chitosan hydrolysis using different chitosanases

To characterize the different chitosanases, enzymes were heterologously expressed and subsequently purified via affinity chromatography (Supplementary Fig. 1). The substrates consisted of a series of well-characterized chitosans with different fractions of acetylation (0.11, 0.19, 0.35, and 0.50). As they were produced from a fully deacetylated parent polymer by partial chemical re-*N*-acetylation, all chitosans had a similar DP (1000–1300; dispersity Đ = 1.7–2.5) and random patterns of acetylation^34^. These chitosans were hydrolyzed for 48 h using the model chitosanases CSN-174, CSN-7M, CSN-MN, or CSN-MHKI until the maximum degree of cleavage was reached. To check if hydrolysis was complete, fresh enzyme was added to the hydrolyzates after 46 h; this led to no further increase of reducing ends for all enzymes at all F_*A*_ (Supplementary Fig. 2). As expected from the literature^20,24,29,35,36^, the chitosanases’ cleavage efficiencies were strongly influenced by the substrate’s F_*A*_ (Supplementary Fig. 3). An increase in substrate F_*A*_ led to reduced activities and was accompanied by a shift in the distribution of the products’ DP from smaller to larger oligomers. In accordance with prior studies^24^, this effect was least pronounced for CSN-174.

### Subsite preferences inferred from sequenced paCOS

To gain insight into the patterns of acetylation of the oligomers produced by the chitosanases, the hydrolysis products obtained from chitosan polymers with different F_*A*_ were analyzed by quantitative sequencing (for detailed oligomer compositions of each hydrolyzate, Supplementary Tables 1–4). The units at the reducing and nonreducing ends of chitosan oligosaccharides directly represent the sugar moieties that had been bound at the active site of an enzyme during cleavage. The two terminal units at the reducing end of an oligomer had occupied subsites (−2) and (−1), whereas the two terminal units at the nonreducing ends had been bound at subsites (+1) and (+2). The molar fractions of each PA of the two terminal units at both reducing and nonreducing ends were determined.

**Fig. 2:**
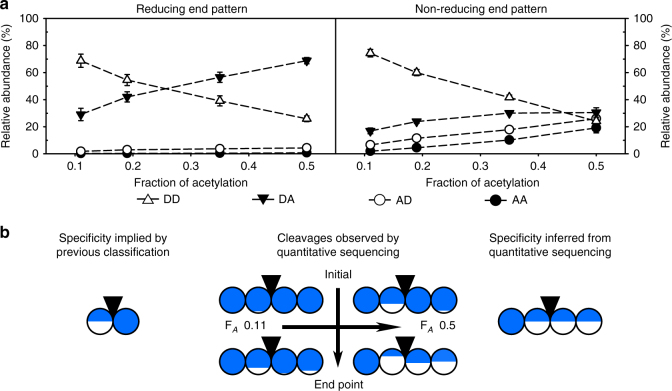
Specificity of the representative class I chitosanase CSN-174 inferred from its products. (**a**) The patterns of acetylation of the two terminal sugar units were analyzed for different F_*A*_ of the substrates by quantitative sequencing of the products at the endpoint of enzymatic hydrolysis. The molar fractions of all possible diads (DD, DA, AD, and AA) are shown. (**b**) The specificity of class I chitosanases implicated by the established qualitative classification system compared to the specificity of CSN-174 (a model class I chitosanase) inferred from the cleavages observed by the quantitative sequencing of its products’ sugar moieties. Diad frequencies at the early time points of enzymatic hydrolysis are taken from Supplementary Table 5. Detailed oligomer compositions are shown in Supplementary Table 1. The circles are divided according to the percentage of the relative abundances (molar fractions) of GlcN (blue) and GlcNAc (white) at the two corresponding subsites, left, (−2) and (−1), and right, (+1) and (+2), of the catalytic cleavage site (indicated by a black inverted triangle). The mean values with standard deviations of at least three independent measurements of three independent enzyme batches are shown (Supplementary Tables 1 and 5)

**Fig. 3:**
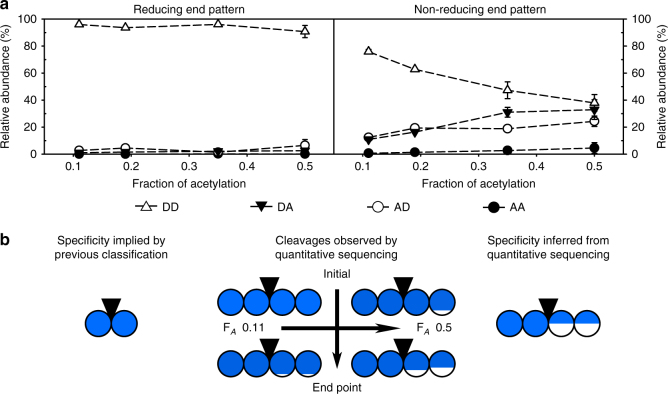
Specificity of the representative class II chitosanase CSN-7M inferred from its products. (**a**) The patterns of acetylation of the two terminal sugar units were analyzed for different F_*A*_ of the substrates by quantitative sequencing of the products at the endpoint of enzymatic hydrolysis. The molar fractions of all possible diads (DD, DA, AD, and AA) are shown. (**b**) The specificity of class II chitosanases implicated by the established qualitative classification system compared to the specificity of CSN-7M (a model class II chitosanase) inferred from the cleavages observed by quantitative sequencing of its products’ sugar moieties. Diad frequencies at the early time points of enzymatic hydrolysis are taken from Supplementary Table 5. Detailed oligomer compositions are shown in Supplementary Table 2. The circles are divided according to the percentage of the relative abundances (molar fractions) of GlcN (blue) and GlcNAc (white) at the two corresponding subsites, left, (−2) and (−1), and right, (+1) and (+2), of the catalytic cleavage site (indicated by a black inverted triangle). The mean values with standard deviations of at least three independent measurements of three independent enzyme batches are shown (Supplementary Tables 2 and 5)

**Fig. 4:**
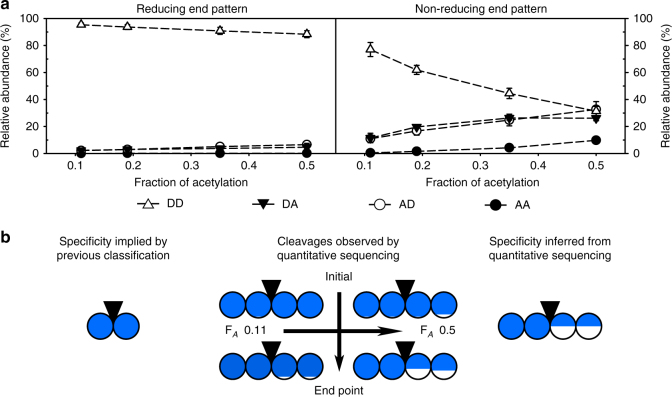
Specificity of CSN-MN (being highly similar to CSN-7M representing class II) inferred from its products. (**a**) The patterns of acetylation of the two terminal sugar units were analyzed for different F_*A*_ of the substrates by quantitative sequencing of the products at the endpoint of enzymatic hydrolysis. The molar fractions (mean values of at least three independent measurements of three independent enzyme batches) of all possible diads (DD, DA, AD, and AA) are shown. (**b**) The specificity of class II chitosanases implicated by the established qualitative classification system compared to the specificity of CSN-MN (97% similar to the model class II chitosanase CSN-7M) inferred from the cleavages observed by the quantitative sequencing of its products’ sugar moieties. Diad frequencies at the early time points of enzymatic hydrolysis are taken from Supplementary Table 5. Detailed oligomer compositions are shown in Supplementary Table 3. The circles are divided according to the percentage of the relative abundances (molar fractions) of GlcN (blue) and GlcNAc (white) at the two corresponding subsites, left, (−2) and (−1), and right, (+1) and (+2), of the catalytic cleavage site (indicated by a black inverted triangle). The mean values with standard deviations of at least three independent measurements of three independent enzyme batches are shown (Supplementary Tables 3 and 5)

**Fig. 5:**
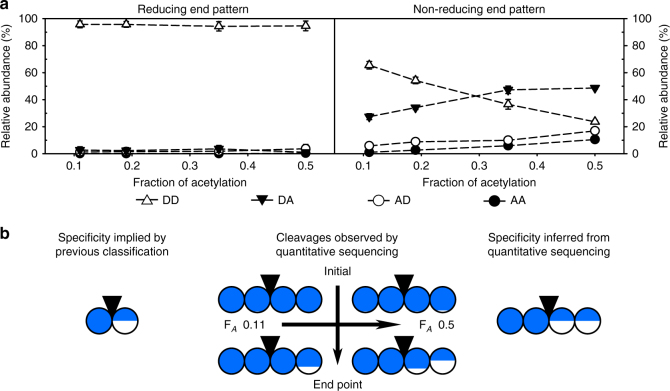
Specificity of the representative class III chitosanase CSN-MHKI inferred from its products. (**a**) The patterns of acetylation of the two terminal sugar units were analyzed for different F_*A*_ of the substrates by quantitative sequencing of the products at the endpoint of enzymatic hydrolysis. The molar fractions (mean values of at least three independent measurements of three independent enzyme batches) of all possible diads (DD, DA, AD, and AA) are shown. (**b**) The specificity of class III chitosanases implicated by the established qualitative classification system compared to the specificity of CSN-MHKI (a model class III chitosanase) inferred from the cleavages observed by quantitative sequencing of its products’ sugar moieties. Diad frequencies at the early time points of enzymatic hydrolysis are taken from Supplementary Table 5. Detailed oligomer compositions are shown in Supplementary Table 4. The circles are divided according to the percentage of the relative abundances (molar fractions) of GlcN (blue) and GlcNAc (white) at the two corresponding subsites, left, (−2) and (−1), and right, (+1) and (+2), of the catalytic cleavage site (indicated by a black inverted triangle). The mean values with standard deviations of at least three independent measurements of three independent enzyme batches are shown (Supplementary Tables 4 and 5)

As observed in the time course of degradation (Supplementary Fig. 2) and previously described in the literature^20^, the degradation proceeded in different phases: an initial phase of fast cleavage faded to an extended slow phase, during which the rest of the possible cleavages occurred. Therefore, the cleavage was analyzed at an initial time point (after 15 min) (Supplementary Table 5) and at the endpoint of cleavage (after 48 h) (Figs. 2–5). While the quantitative endpoint data were quite consistent among enzyme batches, data from the initial phase showed greater variance, as the analyzed fraction of the hydrolyzates (being smaller oligomeric products only) was very small.

### Specificity of class I chitosanase CSN-174

At the endpoint of cleavage, the products of CSN-174 possessed mainly the diads D–D and D–A at the reducing ends (Fig. 2a; Supplementary Table 1). At low fractions of acetylation, there was around twofold more D–D; at higher degrees, there was twofold more D–A. The nonreducing end diads mainly consisted of D–D at low degrees of acetylation. As the abundance of A-units in the substrate increased, all other patterns constantly increased, while the abundance of D–D decreased. The abundance of D–A was always slightly higher than that of A–D, and both of these monoacetylated diads were more abundant than those of A–A. Surprisingly, these patterns indicate that CSN-174 can at least cleave the motifs D–D│A–D and D–D│A–A within the polymeric substrates, and possibly even D–A│A–A (position of cleavage indicated by “│”) (Fig. 2b). In the initial phase, this enzyme almost exclusively hydrolyzed D–D│D–D and D–A│D–D (Supplementary Table 5), thus representing the motifs that are cleaved with the highest velocity; conversely, the other patterns were cleaved much more slowly. The previous classification of CSN-174 as a class I chitosanase (cleaving D│D and A│D^18^) was based on the analysis of a chitosan hydrolyzate with F_*A*_ 0.25–0.35 (Fig. 2b). Similarly in our study, at fractions of acetylation ≤0.35 and especially in the initial phase of cleavage, the specificity of CSN-174 could be mistaken for a specificity that fits class I. Strikingly, after prolonged cleavage of substrates with fractions of acetylation ≥0.35, the enzyme also cleaved the motifs D–D│A–D and D–D│A–A, which conflicts with its classification as class I chitosanase. The products at these conditions rather suggest that CSN-174 has a specificity that fits class IV, as it can cleave all patterns with the likely exception of A│A. Interestingly, the (−2) position of the active site was apparently invariably occupied by a GlcN unit. The (−2) subsite, thus, is the only one showing an absolute specificity for GlcN, while the other subsites only showed preferences for GlcN.

### Specificity of class II chitosanase CSN-7M

CSN-7M showed a high preference for D-units (Fig. 3a). The reducing ends of its products always exclusively consisted of the diad D–D (Supplementary Table 2). At low F_*A*_, the greatest portion of the nonreducing ends also consisted of D–D. At higher F_*A*_, the nonreducing ends increasingly contained D–A and A–D; the former diad was always more abundant. Therefore, CSN-7M was able to cleave the motifs D–D│D–A and, surprisingly, also D–D│A–D (Fig. 3b). In the initial phase of cleavage, the only cleavage patterns observed were D–D│D–D and D–D│D–A (Supplementary Table 5); thus, the cleavage of D│D is more efficient than the cleavage of D│A. Moreover, in this case, both (−2) and (−1) appear to be absolutely specific for GlcN units, while the (+1) and (+2) subsites show only strong preferences for GlcN.

The specificity of CSN-7M, being the most frequently mentioned example of a class II enzyme, was previously inferred from the sequences of the oligomer products D_2–4_, D_4_A_1_, and D_5_A_1_^24^. While these oligomers also fit to class II specificity in our study, we found that, surprisingly, the smaller monoacetylated oligomers D_3_A_1_ and D_2_A_1_, which were not examined in the previous study, contained large amounts of ADDD and ADD (Supplementary Table 6). Thus, CSN-7M can also cleave the motif D–D│A–D, classifying it as class III, though the efficiency to cleave this pattern is rather low.

Compared to our study, that previous study^24^ used a higher substrate concentration and a shorter incubation time, which may have resulted in increased substrate inhibition, as was previously observed for other chitosanases^29,30^, and less complete cleavage of the polymeric substrate. This could explain why, in contrast to previously reported results^24^, we found only small amounts of D_4_ and D_5_A_1_, but larger amounts of D_3_A_1_ and D_2_A_1_ in all hydrolyzates produced with CSN-7M. Moreover, prolonged incubation is necessary to observe these products (Fig. 3b), so it does not seem surprising that no cleavage of the oligomer DDADDA was observed after 20 min of incubation in another previous study^17^. Generally, it remains unclear if there is any chitosanase that shows an absolute specificity for D│D, as required for classifying it as a class II chitosanase.

### Specificity of CSN-MN that is highly similar to CSN-7M

CSN-MN and CSN-7M show 97% identity on the amino acid level, but CSN-7M was previously classified as class II enzyme, while we observed products of CSN-MN indicating that it has class III cleavage specificity (Supplementary Table 3). Interestingly, our data show that CSN-7M is not restricted to cleaving bonds between D│D, but can also cleave D│A. Moreover, we show that the specificity of CSN-MN (Fig. 4) is almost identical to that of CSN-7M (Fig. 3). This finding supports our observation that CSN-7M is also able to cleave D│A. Furthermore, it highlights the precision of quantitative sequencing, as almost identical enzymes were observed to show almost identical specificity.

### Specificity of class III chitosanase CSN-MHKI

The reducing ends formed by CSN-MHKI (Fig. 5a) exclusively consisted of the diad D–D (Supplementary Table 4). Therefore, these subsites seem to be absolutely specific for D-units, like those of CSN-7M and CSN-MN. For the nonreducing ends, a similar trend was observed, as seen for the reducing ends of paCOS produced with CSN-174. At low fractions of acetylation, the nonreducing ends of CSN-MHKI products were present as D–D and D–A, in which the former diad was twofold more abundant. With increasing F_*A*_ of the substrate, the abundance of D–D decreased and the abundance of D–A increased. The abundance of A–D and A–A increased only slightly. In the initial phase of hydrolysis, both the reducing and the nonreducing ends consisted of D-units (Supplementary Table 5). Thus, the cleavage of the motif D–D│D–D was much more efficient than the cleavage of other motifs, and D–D│D–A is highly preferred to D–D│A–D and D–D│A–A (Fig. 5b). Again, as for the previous two enzymes, CSN-7M and CSN-MN, the subsites (−2) and (−1) showed an absolute specificity for GlcN, while the subsites (+1) and (+2) showed only strong preferences for GlcN.

### Boundary between chitosanases and chitinases

**Fig. 6:**
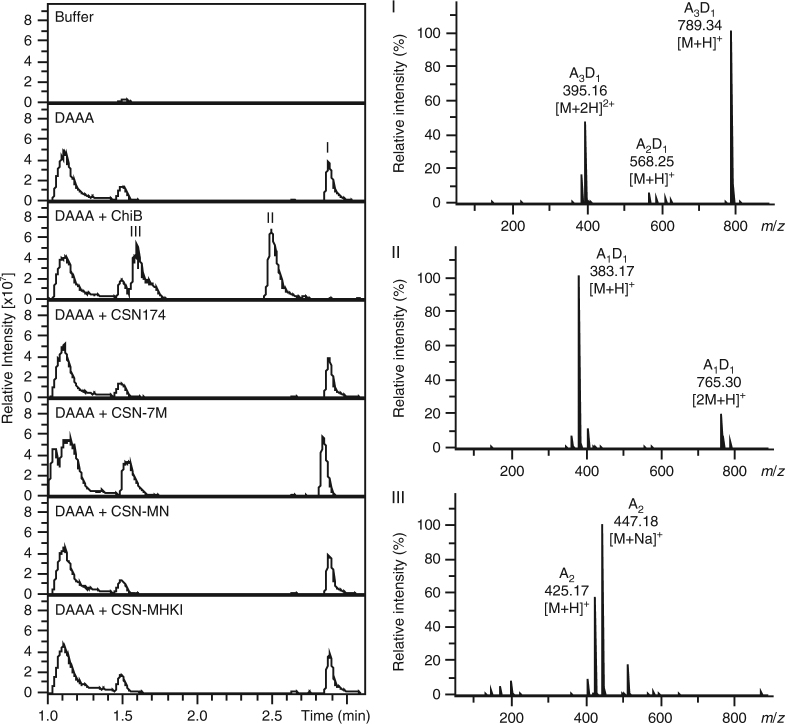
UHPLC-ESI-MS analysis of the hydrolysis of the oligomer DAAA using different chitosanases and a chitinase. The base peak chromatograms (left) and MS spectra belonging to peaks I–III (right) are shown. DAAA was incubated with the chitosanases (CSN-174, CSN-7M, CSN-MN, and CSN-MHKI) and chitinase B (ChiB) from *Serratia marcescens*. While ChiB completely hydrolyzed the tetramer DAAA (Peak III) into the dimers AA (Peak I) and DA (Peak II), no activity was observed for any of the chitosanases

The enzyme nomenclature of chitinases (EC 3.2.1.14) and chitosanases (EC 3.2.1.132) postulates that chitinases cleave A│A and, hence, can cleave the oligomer AAAA, while chitosanases are not able to cleave this pattern. Instead, chitosanases cleave D│D and, hence, can cleave the oligomer DDDD, which chitinases cannot. Yet, our results raise the question: Can CSN-174 cleave the linkage between two A-units? As position (−2) must be occupied by GlcN, the chitin tetramer AAAA or the motif A–A–A–A within a polymer will not be cleaved; however, D–A│A–A may be a potential substrate for this chitosanase. If so, the enzymatic ability of this chitosanase would conflict with the general boundary between chitinase and chitosanase nomenclature, and with its classification as (according to our results) a class IV chitosanase. To investigate this potential conflict, we specifically deacetylated a chitin tetramer at the nonreducing end using the regioselective chitin oligomer deacetylase NodB (yielding the oligomer DAAA)^37^. This oligomer was incubated for 48 h with the chitosanases and, as a comparison, with chitinase B (ChiB) from *Serratia marcescens* ^31^. Subsequently, cleavage was evaluated by ultra-high-performance liquid chromatography electrospray ionization mass spectrometry (UHPLC-ESI-MS; base peak chromatogram and MS spectra corresponding to the substrate and products are shown in Fig. 6). While ChiB cleaved the tetramer DAAA into the dimers DA and AA, no activity was observed for the chitosanases. Therefore, it seems to be unlikely that the motif D–A│A–A can be cleaved by CSN-174, suggesting that the diad A–A found at the nonreducing end of oligomeric products can be produced exclusively from the motif D–D│A–A. Thus, chitosanase CSN-174, like the class IV chitosanase from *Pseudomonas* sp. A-01^17^, cannot productively bind two A-units at subsites (−1) and (+1), while a single A-unit bound at either (−1) or (+1) can be cleaved. However, CSN-174 has six subsites ranging from (−4) to (+2)^38^, so glycosyl residues bound to the additional two subsites could have a stabilizing effect during the enzyme–substrate interaction needed to hydrolyze the motif D–A│A–A.

## Discussion

Our detailed and quantitative data suggest that the current chitosanase classification system is generally too simplistic, as it is based on the enzyme’s fundamental ability to cleave a certain sequence motif within a substrate but is based on specificities of subsites (−1) and (+1) only, and ignores the quantitative efficiency with which a pattern is cleaved. Thus, it cannot properly resolve the substrate specificity or preference of a chitosanase, its impact on products, and finally the differences between different chitosanases. Moreover, our results indicate that CSN-174 and CSN-7M are less specific than those expected from literature^18,24^, and concerning the established classification system, they were in the wrong classes. Thus, the behavior of chitosanases previously classified with this system must be regarded with suspicion.

The PA associated with chitosanase products produced from substrates with lower fractions of acetylation misleadingly implicates a much higher specificity than that which may be observed at higher fractions of acetylation and longer incubation times, as used in this study. Only under the latter conditions does it become clear that there is a difference between an absolute specificity and a strong preference for a D-unit, which forms the essential criteria for the established chitosanase classification system. While we consider the subsite specificities to be an intrinsic property of an enzyme that does not change over the time of incubation or with different fractions of acetylation of the substrate, the different velocities with which different linkages are hydrolyzed result in biphasic curves, leading to erroneous conclusions when using a single substrate (particularly one with low F_*A*_) or a single incubation time (particularly a short one). Thus, it seems unfeasible to meaningfully compare and classify chitosanases without investigating their products when acting on substrates with different fractions of acetylation and at different time points during incubation.

Fortunately, such classification can be performed easily and for several enzymes in parallel through quantitative sequencing, because this technique is simple and uses minimal amounts of oligosaccharides. Moreover, the enzyme reactions can be performed at low substrate concentrations, which ensures that substrate inhibition will have much less of an influence on the formation of products compared to previous studies. Additionally, the deviations of chitosanase behavior found in this study, as compared to the behavior predicted by the classification system, also arose because quantitative sequencing offers a high resolution of product patterns, as all oligomers of the DP 2–6 present were analyzed without any complex separation procedure and, thus, with minimum losses. This much broader range of analyzed products revealed that it is not possible to infer the specificity from products with only one DP alone. Taken together, the use of quantitative sequencing to analyze chitosanase subsite specificity allowed us to produce data that are more appropriate for comparison and is of higher resolution than data produced from other analysis techniques reported in previous studies.

Three types of enzyme subsites were identified: subsites with an absolute specificity for D-units, those with a strong preference for D-units, and those with weak/no preference for D-units. These subsite types seemed to appear in different combinations in the active sites of CSN-174, CSN-7M, CSN-MN, and CSN-MHKI and greatly influenced the composition of hydrolyzates in terms of the produced oligosaccharides’ degrees of polymerization, fractions of acetylation, and patterns of acetylation. Only two classes of chitosanases were observed in this study: one enzyme (CSN-174) with an absolute specificity for a D-unit at (−2) only, while the other enzymes were observed to be specific for D-units at both (−2) and (−1). Interestingly, as observed for SaCsn75A^20^, subsite (−2) was absolutely specific for D-units in all enzymes tested. Although many chitosanases seem to share this absolute specificity, it does not seem to be a universal property, as, for example, the chitosanase from *Bacillus circulans* WL-12 has been reported to accept both D-units and A-units at that subsite^23^. Subsites (+1) and (+2) were much more flexible, although D-units were preferred in all enzymes, especially at the glycosidic bond to be cleaved (the new nonreducing ends). Still, both subsites were able to productively bind motifs with A-units. Interestingly, this flexibility to cleave motifs with A-units was differently pronounced for all chitosanases tested and could be precisely characterized by quantitative sequencing to reveal even slight differences for highly similar chitosanases like CSN-7M and CSN-MN.

The distinction between chitinase and chitosanase via the fundamental ability to cleave A│A and D│D, respectively, seems to be valid for the enzymes investigated in this study. However, recently, a GH 46 chitosanase from *Streptomyces coelicolor* A3(2) was shown to cleave every potential pattern (D│D, A│D, D│A, and A│A) and even a chitin hexamer, although it preferred to cleave D│D^35^. Therefore, even the boundary between chitosanase and chitinase might be more diffuse than the nomenclature suggests. A criterion for this distinction could be that chitosanases generally prefer D-units and, thus, their activity decreases with increasing F_*A*_ of the substrate, while the opposite is true for chitinases. Overall though, making this distinction based on these enzymes’ fundamental abilities to cleave certain motifs seems to be too strict.

Our data clearly show that the current classification system is no longer tenable, and they tend to suggest that a classification based on absolute subsite specificities alone might not be feasible for chitosanases. In any case, as subsite specificities and pronounced subsite preferences are not restricted to the (−1) and (+1) subsites, a modified classification would have to include additional subsites. More chitosanases will have to be examined in detail before firm conclusions can be drawn, but based on our data, a classification system into two classes with the cleavage specificities DD│dd (class A chitosanases) and Dd│dd (class B chitosanases) might tentatively be proposed, in which “D” stands for an absolute specificity and “d” for a strong preference for GlcN units. It must be kept in mind that the members of these classes will differ in details of their subsite preferences and, hence, in the exact composition of their products. Clearly, more chitosanases need to be analyzed in detail before this classification can be considered to adequately cover their diversity, and it is rather likely that more classes will have to be added and/or subclasses will be defined with time when more enzymes are being investigated.

Another way to classify chitosanases, namely into GH families, is based on the overall structural similarity. The two GH 8 chitosanases CSN-7M and CSN-MN share an (α/α)_6_ fold and show an almost identical specificity. Similarly, the GH 46 enzyme CSN-174 shares the α-helical fold of the lysozyme superfamily with the chitosanases from *Pseudomonas* sp. A-01 and *Amycolatopsis* sp. CsO-2, and the three enzymes show similar specificities. However, while the GH 46 enzyme CSN-MHKI is structurally similar to CSN-174^31^, its products are much more similar to those of the GH 8 enzymes CSN-7M and CSN-MN^25^. Thus, in general, similarity of the overall structure and, hence, classification into the GH families, is not closely correlated to specificity. The two GH 8 enzymes analyzed belong to class A, while of the two GH 46 enzymes analyzed, one belongs to class A, while the second one belongs to class B.

The three class A chitosanases CSN-7M, CSN-MN, and CSN-MHKI were all able to cleave the motifs D–D│D–D and D–D│A–D. Their products are similar in DP and acetylation, but they differ in the abundance of patterns of acetylation at the nonreducing end. Because different patterns of acetylation are thought to produce different bioactivities, the paCOS products from these three chitosanases could result in differently accentuated activities, even though their general activity will most likely be similar. Therefore, the complementing information this study offers (about the cleavage sites of four sugar units and their quantitative contribution to the hydrolyzate composition) represents a valuable advance for the reliable production of paCOS with desired functions.

We strongly believe that these findings are also important for other polysaccharides and their degrading enzymes, in particular enzymes acting on other binary polysaccharides, i.e., linear polysaccharides consisting of two different glycosyl units, such as alginates, hyaluronates and glycosaminoglycans, or some pectins. As an example, pectic homogalacturonans from plant cell walls consist of a linear chain of α-1,4-linked galacturonic acid (GalA) residues that can be methyl esterified (GalAMe)^39^. Like chitosans which can have different fractions and patterns of acetylation, homogalacturonans can have different degrees and patterns of methyl esterification (DM and PM, resp.), and these are known to crucially influence their physiological roles, their physicochemical behavior, and their uses in biotechnology or as a functional food additive^40–42^. Like chitinases and chitosanases, homogalacturonan-degrading enzymes are i.a. classified according to their preferred substrate, namely low-DM pectates for endopolygalacturonases (E.C. 3.2.1.15) and endopectate lyases (E.C. 4.2.2.2), and high-DM pectins for endopectin lyases (E.C. 4.2.2.10). These enzymes, their polymeric substrates, and their oligomeric products play important roles in plant growth and development, as well as in plant–pathogen interactions and defence^43–48^. Accordingly, these enzymes and their subsite specificities have been characterized extensively, including by MS analysis of their oligomeric products. As an example, endopolygalacturonase II (PGII) from *Aspergillus niger* cleaved exclusively between two GalA units^49^, while pectin lyase A (PLA) from *A. niger* preferred to cleave between two GalAMe units, but also produced oligomers with GalA at either the reducing or nonreducing end^50^. As a proof of principle that an analysis as described here for chitosanases can also reveal insights into pectin-degrading enzymes, we have performed initial assays to analyze the subsite specificities of one endopolygalacturonase (PG) and one endopectate lyase (PL) by mass spectrometric sequencing of their oligomeric products (Supplementary Fig. 4). Both enzymes turned out to require non-methyl-esterified GalA units at the (−1) and (+1) subsites, and PG but not PL also required GalA at the (+2) position. All other subsites from (−3) to (+3) of both enzymes accepted both GalA and GalAMe units. Of course, in pectins, the situation will be further complicated by the possible presence of additional acetyl groups on *O-*2 or *O-*3 of GalA and/or GalAMe which in turn can influence the activity of the enzymes^51–53^, but this can also be analyzed using MS. Similarly, for enzymes degrading other linear polysaccharides such as glycosaminoglycans consisting of *O*-sulfated or non-*O*-sulfated, and *N*-acetylated or *N*-sulfated residues, or alginates consisting of guluronic and mannuronic acid residues in blockwise or alternating patterns, similar strategies may be developed, based on but not limited to detailed mass spectrometric analyses of their products.

The results of this work highlight the importance of reassessing the current classification system for chitosanases and other polysaccharide-degrading enzymes that are based on subsite specificities, especially because the pattern of substitution—such as acetylation in chitosans or methyl esterification in pectins—of both the polymeric substrates and the oligomeric products has been shown to be a highly important property for reproducible bioactivities. Therefore, the results of this study are fundamental for an in-depth understanding of physiological roles of polysaccharide sequences and for fine-tuning the effective production processes of defined products for reliable applications.

## Methods

### Step-by-step procedure

In this study, all enzymes used were heterologously expressed and purified via affinity chromatography. Afterward, chitosans with different fractions of acetylation were hydrolyzed with the chitosanases. The resulting paCOS were tested for their distribution of the DP via size-exclusion chromatography. Finally, the products of the initial phase and at the endpoint of hydrolysis were characterized by quantitative sequencing, revealing the abundances of acetylation patterns at the terminal regions of the paCOS. These sugar units represent the oligomer parts that were bound in the active site during cleavage. Thus, these quantitative data were used to infer the substrate specificity of the corresponding chitosanases used for their production. Afterward, cleavage of the specific oligomer DAAA was examined to compare the chitosanases with a chitinase.

### Generation of plasmids

All enzymes used were purchased from Thermo Scientific (Schwerte, Germany). The plasmids pET-22b::ChiB-StrepII^31^, pET-22b::StrepII-CSNMN^32^ and pET-22b::NodB-StrepII^37^, as well as their corresponding storage and production strains were used. The coding sequences for CSN-MHKI from *B*. *circulans* MH-K1 (GenBank ID D10624.2) and CSN-7M (GeneBank ID AB051575.1) were optimized for expression in *E*. *coli* and synthesized by GeneArt^®^ (Thermo Scientific, Schwerte, Germany) adding N-terminal *Nde*I and C-terminal *Eco*RI sites. For digestion of DNA, FastDigest^TM^ restriction enzymes were used. Subsequently, the resulting fragments were ligated with pET-22b(+)::StrepIIC^54^ via these two restriction sites using T4 Rapid DNA Ligase, gaining pET-22b::CSN-MHKI-StrepII and pET-22b::CSN-7M-StrepII.

*Csn174* was amplified from pRL270^55^ using the primers CSN174_*Nde*I_fwd (ggaattccATATGCACTCGCAGCACC) and CSN174_*Sma*I_rev (tcccccgggGCTGTTGATGACGTACGG). PCR was accomplished using Phusion Hot Start II High Fidelity proofreading DNA polymerase. The amplicon was digested with *Nde*I and *Sma*I and ligated with pET-22b(+)::StrepIIC digested with *Nde*I and *Eco*ICRI gaining pET-22b::CSN174-StrepII. Primer synthesis and DNA sequencing were accomplished by Eurofins MWG Operon (Eversberg, Germany).

### Heterologous expression and protein purification

*E. coli* DH5α was used for storage of recombinant plasmids. *E*. *coli* Rosetta2 (DE3) [pLysSRARE2] and the original vector pET-22b(+) were used for synthesis of recombinant proteins and purchased from Merck KGaA (Darmstadt, Germany). Transformants of *E*. *coli* DH5α and *E*. *coli* Rosetta2 (DE3) [pLysSRARE2] harboring pET-22b(+)::StrepIIC constructs were grown under selective pressure in LB medium at 37 °C. For long-term storage, *E*. *coli* strains were stored in 0.5 × LB medium containing 25% (v/v) glycerol. For synthesis of recombinant proteins under autoinducing conditions, LB medium was additionally supplemented with medium M (50 × , 2.5 M NH_4_Cl, 1.25 M NaH_2_PO_4_, 1.25 M KH_2_PO_4_, and 0.25 M Na_2_SO_4_) and medium 5052 (50 × , 25% (w/v) glycerol, 10% (w/v) α-lactose monohydrate, and 2.5% (w/v) D-glucose)^56^. The cultures were grown for 6 h at 37 °C and 180 rpm and for additional 42 h at 26 °C and 120 rpm. The cells were harvested by centrifugation for 10 min at 5000 × *g*. The cell pellet was resuspended in 30 ml of FPLC washing buffer (20 mM triethanolamine, 400 mM NaCl, pH 8.0). Cell lysis was induced by freeze-thawing. Two ml of high-salt buffer (1 M triethanolamine, 1 M NaCl, pH 8) were added before the cells were sonicated with a Digital Sonifier® Model 250-D from Branson Ultraschall (Dietzenbach, Germany). The insoluble fraction was pelleted by centrifugation for 40 min at 4 °C and 40,000 × *g*. The soluble fraction was used for protein purification using 1 ml of Strep-Tactin Superflow Plus cartridges from Qiagen (Hilden, Germany) and an ÄKTApure system from GE Healthcare (Munich, Germany)^32^. The proteins were eluted with 20-column volumes of elution buffer (2.5 mM d-desthiobiotin in washing buffer) and concentrated by ultrafiltration (molecular weight cutoff 10 kDa). Afterward, they were supplemented with 10% (v/v) glycerol and stored at 4 °C.

### SDS-PAGE and western blot

For SDS-PAGE^57^, 6 µg of purified protein was mixed with SDS-loading buffer (0.3 M Tris/HCl, pH 6.8 containing 5% (w/v) SDS, 20% (v/v) glycerol, 0.4% (w/v) bromophenol blue, and 0.4% (v/v) β-mercaptoethanol) and denatured for 10 min at 98 °C. Subsequently, the samples were loaded and separated using a 12% (w/v) polyacrylamide gel. Precision Plus Protein^TM^ All Blue Standard from BioRad Laboratories (Munich, Germany) was used as a molecular weight marker. All proteins in the gel were stained using Coomassie^®^ Brilliant Blue G250 from Serva Electrophoresis GmbH (Heidelberg, Germany). A semidry western blot^58^ was accomplished with a PerfectBlue™ Semi-Dry electro blotter from VWR Peqlab (Darmstadt, Germany). Detection of blotted proteins was accomplished with a Fusion SL chemiluminescence detector from Vilber Lourmat Deutschland GmbH (Eberhardzell, Germany). All proteins were transferred to a nitrocellulose membrane (GE Healthcare, Freiburg, Germany) and Strep-tagged proteins were detected via chemiluminescence with a Strep-Tactin® horseradish peroxidase conjugate (Cat. No. 2-1502-001, Lot. No. 1502-5041) from IBA GmbH (Göttingen, Germany) at a dilution of 1:10,000 in TBS (10 mM Tris/HCl, 150 mM NaCl, pH 7.5) containing 5% (w/v) milk powder. The blotted protein standard was detected via white light. Both detections were directly merged with the FUSION-CAP Software from Vilber Lourmat Deutschland GmbH (Eberhardzell, Germany).

### Chitosan F_*A*_ series

As substrates, a series of chitosans with F_*A*_ values of 0.11, 0.19, 0.35, and 0.50 was prepared via re-*N*-acetylation^59^ starting with chitosan F_*A*_ 0.016 DP 1400 (Đ = 2.1) kindly supplied by Mahtani Chitosan PVT. LTD. (Veraval, India).Two g of chitosan were dissolved with acetic acid in 500 ml of water and were supplemented with 0.8 volumes of propanediol. After stirring for 1 h, the solution was kept at rest for 1 h. Acetic anhydride was dissolved in 0.2 volumes of propanediol and added to the solution. The volume of acetic anhydride (*V*_aa_) was calculated for each desired F_*A*_ with the following formula:

$$V_{{\mathrm{aa}}}\, = \,\frac{2g}{161.58\, {\rm g\,{\rm mol}^{-1}}}\, \times \, 0.9\, \times \, \left( {{\mathrm{F}}_{A\left( {{\mathrm{aim}}} \right)}\, - \,{\mathrm{F}}_{A\left( {\mathrm{start}} \right)}} \right)\, \times \,\left(\frac{{{102.09\,{\rm g}\,{\rm mol}^{-1}}}}{{161,58}}\right)$$

F_*A*(aim)_ is the desired F_*A*_, F_*A*(start)_ is the F_*A*_ of the chitosan used for re-*N*-acetylation, 0.9 is the correction factor for the amount of water in chitosan (around 10%). The solution was stirred overnight at RT and the pH was adjusted to 9–10 with ammonia to precipitate the chitosan. The precipitated chitosan was pelleted for 30 min at 12.000 × *g*. The pellet was washed with water and pelleted again 10 times. Afterward, the chitosan was freeze-dried and the average F_*A*_ was checked by ^1^H-NMR^60^.

The specific oligomer DAAA was generated with NodB from *Rhizobium* sp. GRH2 by deacetylation of GlcNAc_4_ from Megazyme (Bray, Ireland)^36^. For this purpose, 1 mM of GlcNAc_4_ was incubated with 2.5 µM of NodB in 50 mM ammonium carbonate buffer at a pH of 8 for 16 h at 37 °C. Afterward, the sample was filtered using a 3-kDA PES filter (VWR, Darmstadt, Germany).

### Enzymatic hydrolysis reactions

For hydrolysis, enzyme concentrations were adjusted according to their activities in the first 15 min of hydrolysis. To determine the patterns of acetylation at the endpoint of hydrolysis, 1 mg ml^−1^ of chitosan was incubated with 1.3 µM CSN-174, 0.2 µM CSN-7M/CSN-MN, or 1.0 µM CSN-MHKI, in all cases corresponding to 15–20 nkat ml^−1^ enzyme activity in 40 mM sodium acetate at a pH of 6 at 37 °C for 48 h with mild shaking. For size-exclusion chromatography, 10 mg of chitosan was hydrolyzed accordingly. To determine the early time point specificity, substrate concentration was increased to 4 mg ml^−1^, while enzyme concentrations were adjusted to 1.5–2.0 nkat ml^−1^ (0.13 µM CSN-174, 0.01 µM CSN-7M/CSN-MN, and 0.1 µM CSN-MHKI). To test for the hydrolysis of the specific oligomer, 1 mg ml^−1^ of DAAA was incubated with 15–20 nkat ml^−1^ (1.3 µM CSN-174, 0.1 µM CSN-7M/CSN-MN, and 1.0 µM CSN-MHKI) and 0.5 µM of ChiB for 48 h at 37 °C with mild shaking.

### Size-exclusion chromatography

Oligomers were separated according to their DP using a SECcurity GPC System (PSS Polymer Standards Service, Mainz, Germany) with an Agilent 1200 series refractive index detector (Agilent Technologies, Santa Clara, USA). Ten mg of chitosan hydrolyzates with F_*A*_ 0.19 and F_*A*_ 0.50 were filtered using a 0.22-µm syringe filter before being injected into the system. Separation was achieved with a HiLoad™ Superdex™ 30 prep-grade column (Pharmacia, Uppsala, Sweden) at a flow rate of 0.3 ml min^−1^ of 0.15 M ammonium acetate buffer at a pH of 4.5. Data were recorded using the WinGPC UniChrom software (PSS Polymer Standards Service, Mainz, Germany).

### Reducing-end assay

Reducing-end concentrations were determined using 3-methyl-2-benzothiazolinone hydrazone hydrochloride hydrate (MBTH)^61^. Samples produced with CSN-174 were diluted at a ratio of 1:2. Aliquots (40 µl) of each reaction mixture were inactivated with 1 volume of 0.5 M NaOH. After adding 20 µl of 1 mg ml^−1^ dithiothreitol and 20 µl of 3 mg ml^−1^ MBTH, samples were incubated for exactly 15 min at 80 °C in a heating block and directly colored by addition of 80 µl of a coloring reagent (0.5% (w/v) FeNH_4_(SO_4_)_2_) × 12 H_2_O, 0.5% (w/v) sulfamic acid, and 0.25 M hydrochloric acid). Subsequently, the absorption at *λ* = 620 nm was determined using a SpectraMax M2 microplate reader from MTX Lab Systems Inc. (Virgina, USA).

### MS and quantitative sequencing of chitosan oligosaccharides

Mass spectrometric measurements were accomplished via UHPLC-ESI-MS. Measurements were performed using an ultra-high-performance liquid chromatography system (Dionex Ultimate 3000RS UHPLC; Thermo Scientific, Milford, USA) via an Acquity UHPLC BEH Amide column (1.7 µM, 2.1 × 150 mm) in combination with a VanGuard precolumn (1.7 µM, 2.1 × 5 mm), both from Waters Corporation (Milford, USA), coupled to an ESI-MS detector (amaZon speed, Bruker Daltonics, Bremen, Germany). Eluent A consisted of 80% (v/v) acetonitrile, and eluent B consisted of 20% (v/v) acetonitrile, both supplemented with 10 mM NH_4_HCO_2_ and 0.1% (v/v) formic acid. A column oven temperature of 35 °C was used, and mass spectra were determined in a positive mode over a scan range of *m*/*z* 50–2000. The parameters for the electrospray ionization were capillary voltage 4 kV, end plate offset voltage 500 V, nebulizer pressure 1 bar, flow rate of the dry gas 8 l min^−1^, and dry temperature 200 °C. Mass spectra were analyzed using Data Analysis 4.1 software (Bruker Daltonics, Bremen, Germany).

To investigate the hydrolysis of the oligomer DAAA, 1 µl of sample was injected to the UHPLC-ESI-MS. The flow rate was adjusted to 0.8 ml min^−1^. Separation was achieved with the following method (percentages refer to the amount of eluent B): 0–0.8ml isocratic at 0%, 0.8–2.3 ml of a linear gradient from 0 to 50%, 2.3–2.5 ml of a linear gradient from 50 to 0%, and 2.5–3.3ml isocratic at 0%.

For quantitative sequencing of paCOS^31^, 60 µg of paCOS from each hydrolyzate were dried and dissolved in 100 µl of 25 mM NaHCO_3_ in 50% (v/v) MeOH. Re-*N*-acetylation was achieved with 5 µl of [^2^H_6_]acetic anhydride (Sigma-Aldrich, St. Louis, USA) added in five steps, each step followed by an incubation step of 15 min at 30 °C at 1200 rpm. Subsequently, samples were vacuum dried, resuspended in 140 µl of H_2_O, and freeze-dried overnight. As an internal standard for quantitative MS, 10 µg of each oligomer from GlcN_2–6_ were re-*N*-acetylated using [^2^H_6_;^13^C_4_] acetic anhydride (Sigma-Aldrich) to introduce a double isotopic label. For tandem MS, 10 µg of re-*N*-acetylated paCOS were vacuum dried, resuspended in 10 µl of H_2_^18^O (euriso-top, Saint-Aubin, France), and incubated at 70 °C overnight to label the reducing ends.

Two µg of labeled samples were analyzed using HILIC-ESI-MS^n^ analysis. The flow rate was adjusted to 0.4 ml min^−1^. Separation for MS was achieved with the following method (percentages refer to the amount of eluent B): 0–3min isocratic at 0%, 3–23 min of a linear gradient from 0 to 20%, 23–25 ml of a linear gradient from 20 to 75%, 25–26 min isocratic at 75%, 26–27 min of a linear gradient from 75 to 0%, and 27–30 min isocratic at 0%. Separation for tandem MS was achieved with the following method (percentages refer to the amount of eluent B): 0–3 min isocratic at 0%, 3–23 min of a linear gradient from 0 to 30%, 23–25 ml of a linear gradient from 30 to 75%, 25–26 min isocratic at 75%, 26–27 min of a linear gradient from 75 to 0%, and 27–30 min isocratic at 0%. The MS parameters for tandem MS were adjusted to the different target masses of paCOS (Supplementary Table 7).

Mass spectrometric data analysis was performed using scripts written in the Python programming language. For quantification, mass signals of [M+H]^+^ ions of re-*N*-acetylated paCOS with unknown concentration and internal standards (*R* ^∗^_2–6_; *R* ^∗^ = GlcN re-*N*-acetylated with [^2^H_6_;^13^C_4_] acetic anhydride) were integrated over an elution duration of each DP. The amount (in ng) of each DP 2–6 paCOS was determined and molar fractions were calculated. In order to determine the sequence(s) of paCOS and their respective abundances, the re-*N*-acetylated paCOS were labeled with ^18^O at their reducing ends. Subsequently, these paCOS were used for MS–MS and their largest b-ion and all y-ions of each precursor were considered to resolve the relative abundance of each possible sequence. Quantification and sequencing data were combined in order to obtain quantitative sequencing information, which was used to determine the molar fractions of the PA of the two terminal sugar moieties at the reducing and nonreducing ends.

### Enzymatic depolymerization of homogalacturonan pectins

One mg of the pectins PC, PCP, or PAP (Supplementary Table 8) was incubated with the pectin-degrading enzymes (Supplementary Table 9) PG (94.5 nkat ml^−1^) or PL (38.8 nkat ml^−1^) in 600 µl of 20 mM ammonium acetate buffer at a pH of 5 for 24 h at 37 °C. After filtration of the samples using 3-kDA PES filters (VWR, Darmstadt, Germany), the ammonium acetate buffer was removed in vacuo, and the dried samples were dissolved in 40 µl of H_2_O. The samples were analyzed using HILIC-ESI-MS^n^ analysis.

### MS of partially methylated GalA oligosaccharides

Liquid chromatography–mass spectrometry measurements were performed using an ultra-high-performance liquid chromatography system (Dionex Ultimate 3000RS UHPLC; Thermo Scientific, Milford, USA) coupled to an ESI-MS detector (amaZon speed, Bruker Daltonics, Bremen, Germany). Eluent A used for the chromatographic separation consisted of 80% (v/v) acetonitrile, and eluent B consisted of 20% (v/v) acetonitrile. Both eluents contained 10 mM ammonium formate and 0.1% (v/v) formic acid. Samples of 1.5 µl were injected into the system and separated via HILIC (Supplementary Table 10). The flow rate was set to 0.4 ml min^−1^ and a column oven temperature of 60 °C was used. Mass spectra were acquired in a negative mode over a scan range of *m*/*z* 50–2000. The parameters for the electrospray ionization were capillary voltage 4 kV, end plate offset voltage 500 V, nebulizer pressure 1 bar, flow rate of the dry gas 8 l min^−1^, and dry temperature 200 °C. Mass spectra were analyzed using Data Analysis 4.1 software (Bruker Daltonics, Bremen, Germany).

To determine the pattern of methylation, 10 µl of the hydrolyzates were dried in vacuo and afterward incubated with 10 µl of H_2_^18^O for 16 h at 70 °C. The same HILIC-ESI-MS method as described above was used for the pattern analysis of the ^18^O-labeled oligosaccharides. The parameters for the isolation and fragmentation during tandem MS in the amaZon speed were isolation width *m/z* 4, group length *m/z* 5, and fragmentation amplitude 80% in the enhanced fragmentation mode of the trap control software. Data were processed and analyzed with the software Compass DataAnalysis 4.1 (Bruker Daltonics, Bremen, Germany).

### Data availability

The data generated and analyzed during the current study are available from the corresponding author on reasonable request.

## Electronic supplementary material

Supplementary Information
