# Supplementary material for: Reassessment of chitosanase substrate specificities and classification
Source: Nat Commun. 2017 Nov 22;8:1698. doi: 10.1038/s41467-017-01667-1 (PMC5700058; doi:10.1038/s41467-017-01667-1)
Supplement: Supplementary file 1 — Supplementary Information [file 41467_2017_1667_MOESM1_ESM.pdf]

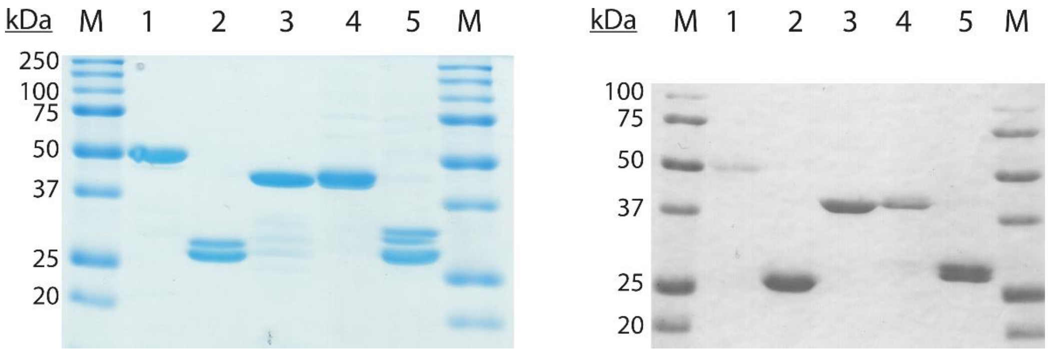

Supplementary Figure 1│SDS-PAGE (left) and corresponding Western-Blot (right) of ChiB (1), CSN-174 (2), CSN-7M (3), CSN-MN (4), and CSN-MHKI (5). The enzymes were synthesized using *E. coli* Rosetta2 (DE3) [pLysSRARE2] production strains carrying the corresponding pET-22b(+)::StrepII constructs. Six µg of purified protein was separated on the gel and stained with Serva Blue G250 (SERVA Electrophoresis GmbH, Germany) or via chemiluminescence with a *Strep*-Tactin® horseradish peroxidase conjugate (IBA, Germany) after blotting. Precision Plus Protein™ All Blue (Bio-Rad Laboratories GmbH, Germany) was used as protein standard (M).

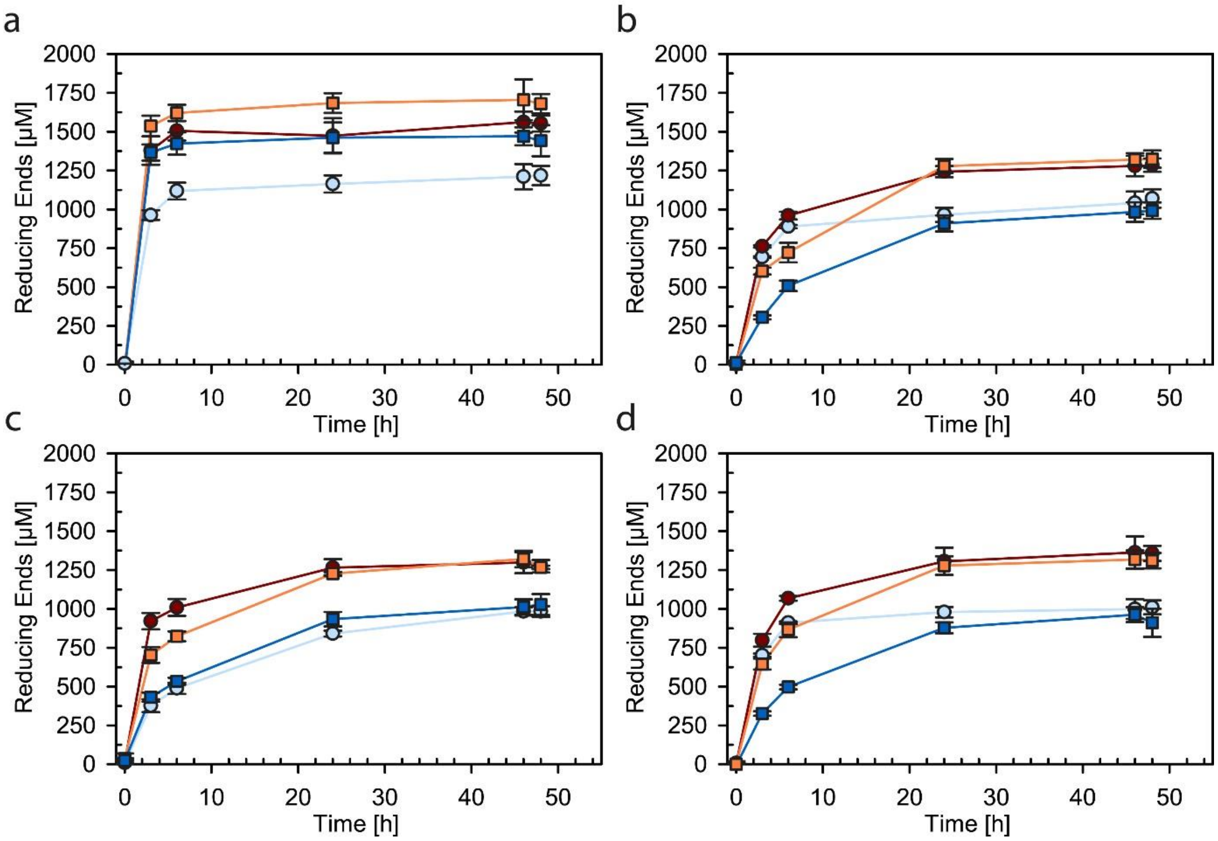

Supplementary Figure 2│Hydrolysis of a series of chitosans (DA 11% (light blue), DA 19% (red), DA 35% (orange), and DA 50% (dark blue)) with (a) CSN-174, (b) CSN-7M, (c) CSN-MN and (d) CSN-MHKI. Samples were incubated for 48 h at 37°C and mild shaking. To check for end-point cleavage, fresh enzyme was added after 46 h. At each time point, reducing end concentrations were determined.

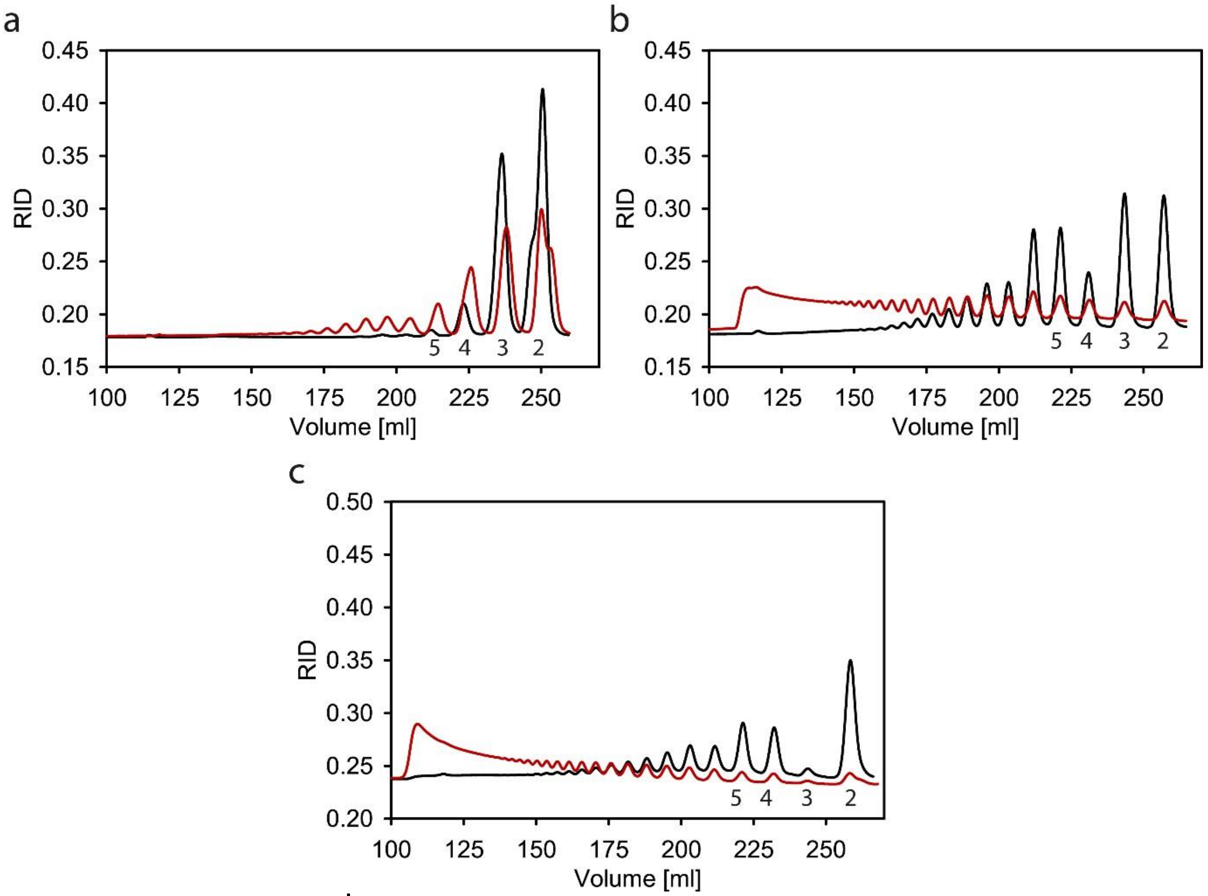

Supplementary Figure 3 | Distribution of the degree of polymerization (DP) of oligomers produced with (a) CSN-174, (b) CSN-MN, and (c) CSN-MHKI. The size distributions of hydrolysates of chitosans with F_A 0.19 (black) and 0.50 (red) were analysed by Size Exclusion Chromatography (SEC) utilizing Refractive Index Detection (RID). For this purpose, the chitosans were hydrolysed with chitosanases until maximal cleavage was reached. Numbers below the peaks indicate the corresponding DPs as verified using UHPLC-ESI-MS. CSN-7M was not analysed, as it shows the same specificity like CSN-MN (compare Figures 3 and 4).

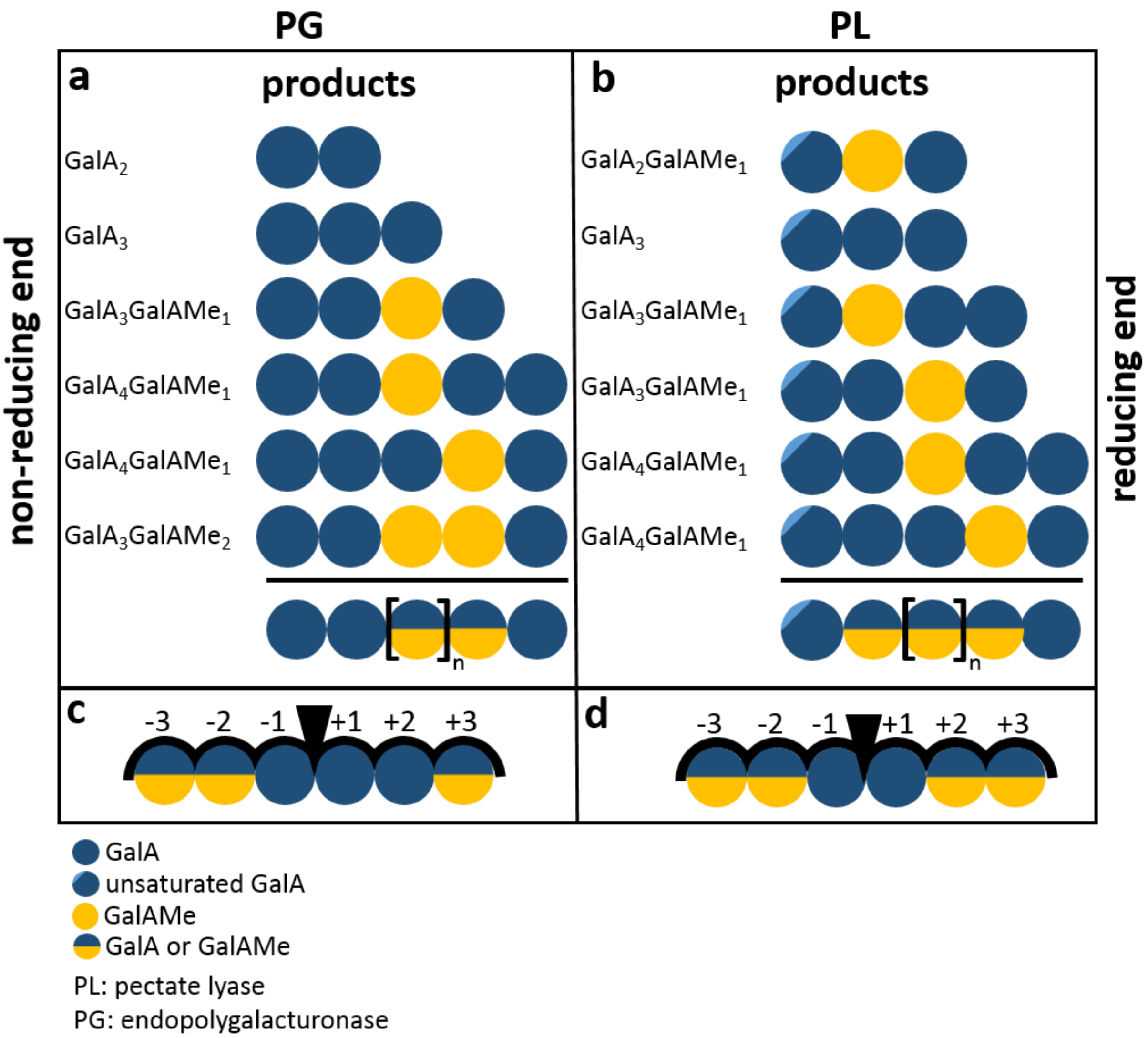

Supplementary Figure 4│Products (up to DP 5) and specificities of an endo polygalacturonidase (PG) from *Aspergillus* sp. and a pectate lyase (PL) from *Aspergillus niger* on partially methyl esterified pectins used as substrates. The products of (a) PG and (b) PL were used to identify the subsite specificities (c and d) of the two enzymes for methyl esterified and non-methyl esterified GalA units in the homogalacturonan chain.

Supplementary Table 1 │ CSN-174 products after the hydrolysis of chitosan with F_A 0.11-0.50. Shown are the molar fractions of each oligomer with an abundance of ≥ 1% and their possible pattern of acetylation. When two or more patterns are given, sequencing of the oligomer was not complete. Data was gained by quantitative sequencing.

F_A	oligomer composition							
	replicate 1		replicate 2		replicate 3		replicate 4	
**0.11**	67.2%	DD	65.1%	DD	56.4%	DD	66.8%	DD
	16.0%	DA	14.2%	DA	19.1%	DA	14.7%	DA
	7.0%	DDA	10.7%	DDA	13.3%	DDA	7.3%	DDA
	3.4%	ADD	3.5%	ADD	3.0%	ADD	2.4%	ADD
	1.2%	AD	1.2%	ADDA	1.5%	AD	2.4%	DDD
	1.1%	ADDA	1.1%	ADA	1.4%	ADA	1.1%	ADDA
	1.1%	ADA			1.2%	ADDA	1.1%	AD
**0.19**	48.4%	DD	47.3%	DD	41.1%	DD	53.1%	DD
	21.8%	DA	20.5%	DA	24.1%	DA	22.9%	DA
	10.0%	DDA	13.5%	DDA	15.0%	DDA	9.3%	DDA
	5.9%	ADD	5.5%	ADD	4.4%	ADD	3.3%	ADD
	2.9%	ADDA<br>DADA	2.8%	ADA	3.3%	ADA	2.6%	ADAD<br>ADDA
	2.8%	ADA	2.3%	ADDA.<br>DADA	2.8%	ADDA	2.5%	ADDA<br>DADA
	2.6%	ADAD<br>ADDA	2.2%	ADAD<br>ADDA	1.8%	AD	2.0%	ADA
	1.7%	AADD	1.5%	AADD	1.6%	AADA	1.4%	AADD
	1.4%	AADA	1.3%	AADA	1.4%	AADD	1.4%	DAD
	1.2%	AD	1.0%	AD	1.0%	DAD	1.4%	AADA
							1.3%	AD
**0.35**	29.8%	DD	31.3%	DD	27.9%	DA	30.3%	DD
	27.3%	DA	27.2%	DA	22.5%	DD	28.3%	DA
	10.1%	DDA	12.3%	DDA	16.7%	DDA	10.7%	DDA
	7.2%	ADD	6.3%	ADD	6.2%	ADA	5.1%	ADD
	5.6%	ADA	4.6%	ADA	6.0%	ADD	4.7%	ADA
	4.2%	ADDA	4.4%	ADDA<br>DADA	5.1%	ADDA	4.6%	ADDA
	3.8%	AADD	4.1%	ADAD<br>ADDA	3.2%	AADA	3.2%	AADA
	3.1%	AADA	2.7%	AADD	2.8%	AADD	2.6%	AADD
	1.4%	AD	2.6%	AADA	1.9%	AD	1.9%	DAD
	1.4%	AADAD<br>AADDA	1.5%	DAD	1.8%	AADDA	1.9%	AADDA
	1.3%	DADA	1.3%	AD			1.5%	AD
	1.3%	AADDA<br>DAADA	1.3%	AADDA			1.2%	AAADD
	1.2%	DAD	1.2%	DAAD<br>DADA			1.1%	AAADA
**0.50**	25.5%	DA	24.6%	DA	26.3%	DA	32.9%	DA
	14.5%	DD	13.4%	DD	12.0%	ADA	17.8%	DD
	12.2%	ADA	12.0%	ADA	11.6%	DDA	10.1%	ADA
	8.3%	AADA	11.2%	DDA	11.4%	DD	8.7%	AADA
	6.9%	ADD	7.2%	AADA	8.0%	AADA	7.7%	DDA
	6.8%	DDA	6.4%	ADD	7.4%	ADAD<br>ADDA	7.2%	ADAD<br>ADDA
	6.7%	ADDA<br>DADA	6.3%	ADDA<br>DADA	7.2%	ADDA<br>DADA	7.0%	ADDA<br>DADA
	6.6%	ADAD<br>ADDA	6.2%	ADAD<br>ADDA	5.3%	ADD	4.7%	ADD
	4.2%	AADD	3.3%	AADD	3.0%	AADD	4.0%	AADD
	4.0%	AAADD<br>AADAD<br>AADDA	2.7%	AAADA	2.9%	AAADA	2.3%	DAD
	3.2%	AAADA	2.4%	AADDA<br>DAADA	2.6%	AADDA	1.5%	ADAD<br>DAAD
	2.6%	AADDA<br>DAADA<br>DADAA	2.0%	AADAD<br>AADDA	1.4%	AAADD	1.5%	AD
	2.0%	AAADD<br>AADAD<br>DAAAD	1.9%	DAD	1.4%	AD	1.3%	DAAD<br>DADA
	1.3%	AD	1.5%	AAADD	1.2%	AAAADA		

D: GlcN; A: GlcNAc.

Supplementary Table 1 | Continued

F_A	oligomer composition							
	replicate 1		replicate 2		replicate 3		replicate 4	
**0.50**	1.0%	AAAADA	1.3%	DAAD<br>DADA				
			1.2%	ADAD.<br>DAAD				
			1.2%	AD				

D: GlcN; A: GlcNAc.

Supplementary Table 2 │ CSN-7M products after the hydrolysis of chitosan with F_A 0.11-0.50. Shown are the molar fractions of each oligomer with an abundance of ≥ 1% and their possible pattern of acetylation. When two or more patterns are given, sequencing of the oligomer was not complete. Data was gained by quantitative sequencing.

F_A	oligomer composition							
	replicate 1		replicate 2		replicate 3		replicate 4	
**0.11**	53.8%	DD	56.1%	DD	57.2%	DD		
	16.1%	DDD	14.5%	DDD	14.5%	DDD		
	6.6%	ADDD	6.1%	ADDD	6.2%	ADDD		
	6.5%	DADDD	5.3%	DADDD	5.6%	DADDD		
	2.8%	ADD	3.3%	DADD	3.0%	ADD		
	2.5%	DADD	3.0%	ADD	3.0%	DADD		
	2.1%	ADADD<br>DAADD	1.9%	DDADD	2.2%	ADADD<br>DAADD		
	1.7%	DDADD	1.9%	ADADD<br>ADDAD	1.6%	DDADD		
	1.5%	ADADD<br>ADDAD	1.0%	ADADDD<br>ADDADD	1.4%	AADDDD<br>ADADDD<br>ADDADD<br>DAADDD<br>DADADD<br>DDAADD		
	1.4%	DDAD			1.4%	DDAD		
	1.0%	ADADDD<br>ADDADD<br>ADDDAD			1.4%	DAADDD<br>DADADD<br>DADDAD<br>DADDDA<br>DDAADD<br>DDADAD<br>DDADDA<br>DDDAAD<br>DDDADA		
					1.3%	ADADD<br>ADDAD		
					1.2%	DAADD<br>DADAD		
**0.19**	43.6%	DD	44.0%	DD	44.8%	DD		
	11.1%	DDD	12.1%	DDD	11.3%	DDD		
	8.7%	ADDD	9.1%	ADDD	8.3%	ADDD		
	7.2%	DADDD	7.7%	DADDD	7.1%	DADDD		
	4.2%	DADD	4.5%	ADADD<br>DAADD	4.8%	DADD		
	3.9%	ADD	3.8%	ADD	4.2%	ADADD<br>DAADD		
	3.9%	DDADD	3.3%	DADD	3.8%	ADADD<br>ADDAD		
	3.5%	ADADD<br>ADDAD	3.2%	DDADD	3.5%	ADD		
	2.6%	ADADDD<br>DAADDD	3.1%	ADADD<br>ADDAD	3.4%	DDADD		
	2.2%	DAADD<br>DADAD	2.5%	DAADD<br>DADAD	2.2%	ADADDD<br>DAADDD		
	1.9%	ADADDD<br>ADDDAD	1.8%	ADADDD<br>DAADDD	2.1%	ADADDD<br>ADDADD<br>ADDDAD<br>ADDDDA		
	1.6%	DAADDD<br>DADDAD	1.7%	DAADDD<br>DADADD<br>DADDAD	1.7%	DAADD<br>DADAD		
	1.3%	DDAD	1.6%	DDAD	1.4%	DAADDD<br>DADADD<br>DADDAD<br>DADDDA		
	1.1%	AADDD	1.2%	ADADDD<br>ADDADD<br>ADDDAD	1.4%	ADDAD<br>DADAD		
			1.1%	ADDADD<br>ADDDAD<br>DADADD<br>DADDAD	1.3%	ADDADD<br>ADDDAD<br>ADDDDA<br>DADADD<br>DADDAD<br>DADDDA		
			1.1%	ADDAD<br>DADAD	1.3%	ADDADD<br>ADDDAD<br>DADADD<br>DADDAD<br>DDAADD<br>DDADAD		
					1.0%	DDAD		

D: GlcN; A: GlcNAc.

Supplementary Table 2 │ Continued

F_A	oligomer composition							
	replicate 1		replicate 2		replicate 3		replicate 4	
**0.35**	20.9%	DADDD	20.5%	DADDD	19.2%	DADDD		
	18.6%	DD	18.7%	DD	17.8%	DD		
	12.2%	ADDD	12.1%	ADDD	17.8%	DDD		
	10.5%	DDD	10.2%	DDD	9.7%	ADDD		
	7.0%	DDADD	6.8%	DDADD	5.7%	DDADD		
	4.9%	DADD	5.5%	ADADD<br>DAADD	4.5%	ADADDD<br>DAADDD		
	3.9%	DAADDD<br>DADADD	4.1%	DADD	4.4%	DDADDD		
	3.8%	ADADDD<br>DAADDD	4.1%	ADADDD<br>DAADDD	4.0%	AADDD<br>ADADD<br>DAADD		
	3.3%	DDADDD	3.5%	DAADD<br>DADDA	3.3%	DAADDD<br>DADADD		
	3.2%	ADADD	3.1%	DDADDD	3.1%	ADDDD		
	3.0%	DAADD	3.0%	AADDD	3.0%	DAADD<br>DADAD<br>DADDA<br>DDAAD<br>DDADA		
	2.2%	AADDD	2.6%	ADADD<br>ADDDA	2.5%	DDDD		
	1.6%	ADD	2.6%	DAADDD<br>DADADD<br>DADDAD	2.4%	DADD		
	1.5%	DDDDD	2.1%	ADADDD<br>ADDADD<br>ADDDAD	2.1%	AADDD<br>ADADD<br>ADDAD<br>ADDDA		
	1.1%	ADDADD<br>DADADD	1.6%	ADD	1.8%	ADADDD<br>ADDADD		
	1.1%	ADADDD<br>ADDADD	1.5%	DDDDD	1.4%	ADD		
					1.0%	DDDDD		
**0.50**	17.0%	DADDD	20.7%	DADDD	17.1%	DADDD		
	13.6%	ADDD	14.2%	DD	15.4%	DD		
	13.4%	DD	12.0%	ADDD	12.1%	ADDD		
	7.9%	DAADD	10.3%	DDD	8.9%	DDD		
	5.8%	DAADDD<br>DADADD<br>DADDAD<br>DADDDA	8.9%	ADADDD<br>DAADDD	6.7%	ADADDD<br>DAADDD		
	5.4%	ADADDD<br>DAADDD	7.0%	DAADDD<br>DADADD	5.3%	DADD		
	5.4%	DADD	6.9%	AADDD<br>ADADD<br>ADDAD<br>ADDDA	4.7%	DAADD		
	5.0%	DDD	6.6%	AADDD<br>ADADD<br>DAADD	4.1%	DAADDD<br>DADADD		
	4.7%	DDADD	4.5%	DDADD	3.9%	ADADDD<br>ADDADD		
	4.4%	ADADD	3.9%	DAADD<br>DADAD<br>DADDA<br>DDAAD<br>DDADA	3.7%	AADDD		
	3.9%	AADDD	3.5%	DADD	3.7%	DDADD		
	2.6%	ADD	3.3%	ADDAD<br>DADAD<br>DDAAD	3.4%	ADADD		
	2.4%	DDADDD	2.6%	DDADDD	3.4%	ADD		
	2.0%	ADDADD<br>ADDDAD<br>DADADD<br>DADDAD<br>DDAADD<br>DDADAD	2.6%	ADADDD<br>ADDADD	2.1%	DDADDD		
	2.0%	ADDADD<br>ADDDAD<br>ADDDDA<br>DADADD<br>DADDAD<br>DADDDA	2.6%	ADD	1.3%	ADDADD<br>DADADD		
	1.7%	AAADDD	1.1%	DDDDD	1.1%	ADDDD		
	1.6%	ADADDD<br>ADDADD<br>ADDDAD<br>ADDDDA	1.0%	AAADDD<br>AADADD<br>ADAADD	1.1%	DDDDD		

D: GlcN; A: GlcNAc.

Supplementary Table 2 | Continued

F_A	oligomer composition							
	replicate 1		replicate 2		replicate 3		replicate 4	
**0.50**	1.5%	DDDDD	1.0%	DDDD	1.0%	ADAADD<br>ADADAD<br>ADADDA<br>DAAADD<br>DAADAD<br>DAADDA		
	1.1%	AADD						
	1.0%	DDDAD						
	1.0%	AAADD						

D: GlcN; A: GlcNAc.

Supplementary Table 3 │CSN-MN products after the hydrolysis of chitosan with F_A 0.11-0.50. Shown are the molar fractions of each oligomer with an abundance of ≥ 1% and their possible pattern of acetylation. When two or more patterns are given, sequencing of the oligomer was not complete. Data was gained by quantitative sequencing.

F_A	oligomer composition							
	replicate 1		replicate 2		replicate 3		replicate 4	
**0.11**	37.8%	DD	35.2%	DDD	39.5%	DD	49.3%	DD
	28.1%	DDD	33.2%	DD	25.0%	DDD	30.8%	DDD
	9.2%	ADDD	9.8%	DADDD	8.5%	ADDD	7.2%	ADDD
	8.7%	DADDD	5.3%	ADDD	6.6%	DADDD	3.8%	DADD
	2.6%	DDADD	4.0%	DDADDD	4.0%	DADD	3.3%	DDA
	2.3%	DADD	2.1%	DADD	2.6%	DDADD	1.3%	ADD
	1.9%	DDADDD	2.0%	DDADD	2.6%	ADD		
	1.6%	ADADDD<br>DAADDD	1.2%	ADADDD<br>DAADDD	1.7%	ADADDD<br>DAADDD		
	1.4%	ADD	1.1%	DDA	1.6%	DAADDD<br>DADADD		
	1.3%	DAADDD<br>DADDAD	1.0%	DAADDD<br>DADDAD<br>DADDDA	1.1%	DDADDD		
	1.0%	ADADD<br>ADDAD			1.1%	ADADD<br>ADDAD<br>ADDDA		
					1.0%	ADADD<br>DAADD		
**0.19**	29.4%	DD	27.5%	DDD	32.9%	DD	32.7%	DD
	21.4%	DDD	27.4%	DD	18.5%	DDD	21.2%	DDD
	11.5%	ADDD	13.4%	DADDD	13.7%	ADDD	14.7%	DADDD
	11.3%	DADDD	9.0%	ADDD	10.4%	DADDD	10.5%	ADDD
	3.9%	DADD	4.7%	DDADDD	4.8%	DADD	3.7%	DDADD
	2.5%	ADADDD<br>DAADDD	3.0%	DDADD	4.5%	ADADD<br>DAADD	3.1%	DADD
	2.2%	ADD	2.5%	DADD	3.7%	ADD	1.8%	DDA
	2.1%	DDADDD	2.5%	ADADDD<br>DAADDD	2.6%	ADADD<br>ADDDA	1.7%	ADDAD<br>DADAD
	2.1%	ADADDD<br>ADDADD<br>ADDDDA	1.9%	DAADDD<br>DADADD	2.5%	DDADD	1.6%	DAADD<br>DADAD<br>DADDA
	2.0%	DDADD	1.5%	ADADD<br>DAADD	2.3%	DAADD<br>DADDA	1.5%	AADDD
	2.0%	DAADDD<br>DADADD<br>DADDDA	1.1%	ADD	1.4%	DDAD	1.4%	ADADD<br>ADDAD<br>ADDDA
	1.8%	ADADD<br>DAADD	1.0%	AADDD	1.0%	DAD	1.3%	ADD
	1.7%	DAADD<br>DADAD<br>DADDA	1.0%	DDDDA			1.0%	DDDDD
	1.4%	ADADD<br>ADDAD<br>ADDDA	1.0%	ADADD<br>ADDDA				
	1.3%	DDAD	1.0%	ADADDD<br>ADDADD				
	1.1%	ADDADD<br>DADADD						
	1.0%	AADDD						
	1.0%	DDDAD						
	1.0%	ADDAD<br>DADAD						
**0.35**	20.4%	DD	20.3%	DD	20.8%	DD	23.5%	DD
	16.4%	ADDD	17.0%	DDD	14.3%	ADDD	16.2%	DADDD
	11.3%	DADDD	15.7%	DADDD	11.4%	DADDD	14.5%	ADDD
	11.2%	DDD	13.9%	ADDD	8.9%	DDD	13.0%	DDD
	5.8%	ADADDD<br>DAADDD	5.8%	ADADDD<br>DAADDD	6.3%	DADD	5.6%	ADADD<br>DAADD
	4.2%	DADD	4.3%	DAADDD<br>DADADD	6.1%	ADADD<br>DAADD	5.0%	DAADD<br>DADDA
	3.9%	ADADDD<br>ADDADD<br>ADDDDA	3.9%	DADD	5.7%	ADADDD<br>DAADDD	4.7%	DDADD
	3.6%	DAADDD<br>DADADD<br>DADDDA	3.8%	DDADDD	4.7%	ADADD<br>ADDAD<br>ADDDA	4.2%	DADD
	3.6%	ADD	3.5%	DDADD	4.6%	ADD	3.0%	AADDD
	3.5%	DDADD	3.5%	AADDD	4.2%	DAADDD<br>DADADD<br>DADDAD	2.1%	ADD

D: GlcN; A: GlcNAc.

Supplementary Table 3 │ Continued

F$_A$	oligomer composition							
	replicate 1		replicate 2		replicate 3		replicate 4	
0.35	3.2%	ADADD<br>DAADD	2.6%	ADADDD<br>ADDADD	3.3%	DAADD<br>DADAD<br>DADDA	2.1%	ADADD<br>ADDDA
	3.2%	DAADD<br>DADAD	2.4%	ADADD<br>DAADD	3.0%	ADADDD<br>ADDADD<br>ADDDAD	1.8%	DDA
	3.1%	ADADD<br>ADDAD	2.3%	DAADD<br>DADDA	2.3%	DDADD	1.6%	DDAD
	3.1%	ADDAD<br>DADAD	1.8%	ADD	2.2%	AADDD	1.5%	ADDDA<br>DADDA
	2.9%	AADDD	1.2%	ADDADD<br>DADADD	2.1%	ADDAD<br>DADAD<br>DDAAD	1.4%	DDDDD
	1.8%	ADDADD<br>ADDDDA<br>DADADD<br>DADDDA			1.9%	ADDAD<br>ADDDA<br>DADAD<br>DADDA	1.0%	ADAD<br>ADDA
	1.7%	DDADDD			1.9%	DDAD		
	1.5%	DDAD			1.5%	ADDADD<br>ADDDAD<br>DADADD<br>DADDAD		
	1.5%	ADDADD<br>DADADD<br>DDAADD			1.2%	AADADD<br>AADDAD<br>AADDDA<br>ADAADD<br>ADADAD<br>ADADDA		
	1.4%	ADDDDA<br>DADDDA<br>DDADDA			1.2%	ADAD<br>DAAD		
	1.1%	ADAD<br>ADDA			1.2%	ADDDAD<br>DADDAD<br>DDADAD		
	1.0%	ADAD<br>DAAD			1.0%	ADDADD<br>DADADD<br>DDAADD		
0.50	15.7%	DD	15.2%	ADDD	15.7%	DD	17.4%	ADDD
	15.2%	ADDD	14.1%	DD	15.1%	ADDD	17.4%	DD
	8.9%	ADADD<br>DAADD	13.6%	DADDD	8.4%	DADDD	13.3%	DADDD
	7.7%	ADADD<br>ADDAD	10.7%	DDD	8.0%	ADADDD<br>DAADDD	10.0%	DDD
	7.3%	DADDD	9.2%	ADADDD<br>DAADDD	7.6%	ADADD<br>DAADD	7.6%	DAADD<br>DADAD<br>DADDA
	5.9%	DADD	6.9%	AADDD	6.9%	ADADDD<br>ADDADD	7.0%	AADDD
	5.9%	ADD	5.8%	DAADDD<br>DADADD	6.5%	DADD	6.6%	ADADD<br>DAADD
	5.1%	AADDDD<br>ADADDD<br>ADDADD<br>ADDDDA	4.5%	ADADDD<br>ADDADD	6.0%	DAADD<br>DADAD	4.3%	DADD
	4.8%	DAADDD<br>DADADD<br>DADDDA<br>DDAADD<br>DDADDA	4.2%	DADD	5.8%	ADD	3.1%	ADD
	4.8%	AADDDD<br>ADADDD<br>DAADDD	3.4%	DAADD<br>DADAD<br>DADDA	5.7%	ADADD<br>ADDAD	3.1%	ADADD<br>ADDAD<br>ADDDA
	4.7%	DAADD<br>DADAD	3.0%	DDADDD	4.4%	DDD	2.6%	ADDDA<br>DADDA
	4.7%	DDD	2.8%	ADD	4.3%	AADDD	2.2%	DDA
	4.4%	ADDADD<br>DADADD<br>DDAADD	2.7%	ADADD<br>DAADD	4.1%	ADDAD<br>DADAD	2.0%	DDADD
	4.2%	AADDD	2.0%	ADADD<br>ADDAD<br>ADDDA	3.0%	ADAADD	1.8%	ADDA<br>DADA
	3.5%	ADDAD<br>DADAD	1.9%	DDADD	2.7%	DAADDD<br>DADADD	1.6%	ADAD<br>ADDA
	2.7%	ADAD<br>DAAD	1.7%	ADDAD<br>DADAD	1.9%	AADD	1.6%	AADD
	2.6%	ADAD<br>ADDA	1.5%	AADADD<br>AADDAD<br>ADAADD<br>ADADAD<br>ADDAAD<br>DAAADD<br>DAADAD<br>DADAAD	1.8%	ADAD<br>DAAD	1.5%	ADDAD<br>DADAD
	1.8%	DDAD	1.0%	DDA	1.6%	ADAD<br>ADDA	1.3%	AAADD
	1.7%	ADAADD<br>ADADAD<br>ADADDA	1.0%	ADDDA<br>DADDA	1.6%	ADDADD<br>DADADD	1.1%	DA

D: GlcN; A: GlcNAc.

Supplementary Table 3 | Continued

F_A	oligomer composition							
	replicate 1		replicate 2		replicate 3		replicate 4	
0.50	1.6%	AADADD<br>ADAADD<br>DAAADD	1.0%	AAADDD	1.5%	AAADDD	1.1%	DDAD
	1.5%	DDADD	1.0%	AADADD<br>AADDAD<br>AADDDA	1.4%	DDADD	1.0%	DAAD<br>DADA
	1.3%	AAADDD			1.2%	DDADDD		
	1.1%	AADD			1.1%	DDAADD		
	1.1%	DAAADD<br>DAADAD<br>DAADDA			1.0%	AAADD		
	1.0%	AADDAD<br>ADADAD<br>DAADAD						

D: GlcN; A: GlcNAc.

Supplementary Table 4│CSN-MHKI products after the hydrolysis of chitosan with F_A 0.11-0.50. Shown are the molar fractions of each oligomer with an abundance of ≥ 1% and their possible pattern of acetylation. When two or more patterns are given, sequencing of the oligomer was not complete. Data was gained by quantitative sequencing.

F_A	oligomer composition							
	replicate 1		replicate 2		replicate 3		replicate 4	
**0.11**	61.6%	DD	62.7%	DD	60.1%	DD	65.2%	DD
	13.1%	DADD	12.9%	DADD	11.5%	DADD	9.0%	DADDD
	11.4%	DADDD	11.5%	DADDD	10.9%	DADDD	8.1%	DADD
	3.7%	ADDD	3.3%	ADDD	3.0%	ADDD	2.8%	DA
	2.0%	DAADDD<br>DADADD	2.0%	DAADDD<br>DADADD	2.4%	DAADDD<br>DADADD	2.7%	ADDD
	1.5%	ADD	1.3%	ADD	2.2%	DAADD<br>DADAD<br>DADDA	1.8%	DDD
	1.5%	ADADDD<br>DAADDD	1.3%	ADDADD<br>DADADD	1.5%	ADADDD<br>DAADDD	1.7%	DADDA
	1.3%	ADDADD<br>DADADD	1.1%	ADADDD<br>DAADDD	1.3%	ADDADD<br>DADADD	1.0%	DAADD<br>DADAD
	1.0%	AADDD	1.0%	ADADD<br>DAADD	1.2%	DDADD		
					1.2%	DA		
					1.1%	DAD		
					1.1%	ADD		
**0.19**	48.2%	DD	51.9%	DD	48.7%	DD	53.6%	DD
	18.6%	DADD	17.9%	DADD	12.9%	DADD	13.4%	DADDD
	11.0%	DADDD	12.8%	DADDD	11.2%	DADDD	12.1%	DADD
	5.0%	ADDD	4.2%	ADDD	5.5%	ADDD	4.4%	ADDD
	3.9%	DAADDD<br>DADADD	2.5%	DAADD	3.9%	DAADDD<br>DADADD	3.6%	DAADD<br>DADDA
	2.6%	ADD	2.3%	ADD	3.1%	ADDADD<br>DADADD	2.5%	ADDDA<br>DADDA
	2.3%	ADDADD<br>DADADD	2.1%	AADDD	3.0%	ADD	2.1%	ADADD<br>DAADD
	2.2%	ADADDD<br>DAADDD	1.1%	DDADD	2.5%	ADADD<br>ADDAD<br>DAADD<br>DADAD	1.7%	DA
	2.1%	AADDD			2.2%	DAADD<br>DADAD<br>DADDA	1.7%	AADDD
	1.7%	DAADD<br>DADAD			1.7%	DAD	1.3%	ADD
					1.6%	ADADDD<br>DAADDD	1.0%	DDADD
					1.5%	AADDD	1.0%	ADADD<br>ADDDA
					1.2%	DDADD		
**0.35**	28,5%	DD	33,2%	DD	32,0%	DD	37,2%	DD
	26,7%	DADD	23,0%	DADD	20,4%	DADD	18,7%	DADD
	9,9%	DADDD	11,8%	DADDD	13,5%	DADDD	14,5%	DADDD
	5,5%	DAADDD<br>DADADD<br>DADDDA	5,3%	DAADDD<br>DADADD	6,7%	DAADDD<br>DADADD<br>DADDAD	7,2%	DAADD<br>DADDA
	5,3%	ADDD	5,1%	DAADD<br>DADAD	4,3%	DAADD	5,8%	ADADD<br>DAADD
	4,9%	DAADD	5,0%	ADDD	3,8%	AADDD	4,2%	ADDD
	4,1%	ADD	4,0%	AADDD	3,6%	ADDADD<br>DADADD	3,6%	AADDD
	4,1%	ADADDD<br>DAADDD	3,2%	ADADDD<br>DAADDD	3,4%	ADDD	3,1%	ADDDA<br>DADDA
	3,8%	AADDD	3,2%	ADD	2,9%	ADADDD<br>DAADDD	2,1%	ADD
	2,4%	ADDADD<br>DADADD	2,8%	ADDADD<br>DADADD	2,0%	ADD	1,6%	ADADD<br>ADDDA
	1,8%	ADADDD<br>ADDADD<br>ADDDDA	2,1%	DDADD	1,5%	DADDA	1,4%	DA
	1,5%	AADD	1,2%	AADD	1,4%	DAD	1,3%	AADD
	1,3%	ADADD			1,3%	ADDDAD<br>DADDAD	1,1%	DADA
	1,2%	AADADD<br>ADAADD<br>DAAADD			1,2%	ADADDD<br>ADDADD<br>ADDDAD	1,0%	DDADD
					1,1%	AADD	1,0%	DDDDD
					1,1%	AADADD<br>DAAADD		

D: GlcN; A: GlcNAc.

Supplementary Table 4 | Continued

F_A	oligomer composition							
	replicate 1		replicate 2		replicate 3		replicate 4	
**0.50**	29.5%	DADD	23.1%	DADD	22.6%	DADD	49.5%	DD
	22.2%	DD	19.4%	DD	19.4%	DD	25.7%	DADD
	9.0%	DAADD	7.9%	DAADD	9.8%	DAADD<br>DADAD	5.4%	ADDD
	8.1%	DADDD	7.7%	DADDD	6.8%	DADDD	4.3%	ADD
	7.0%	AADDD	7.4%	DAADDD<br>DADADD	6.7%	ADDD	3.4%	ADDA<br>DADA
	6.3%	ADD	6.6%	ADDADD<br>DADADD	5.3%	DAADDD<br>DADADD<br>DADDAD	3.0%	DA
	5.9%	ADDD	6.2%	ADDD	4.4%	ADD	2.9%	AADD
	4.2%	AADD	6.0%	ADD	3.8%	AADDD	2.2%	ADAD<br>ADDA
	2.6%	ADADD	5.9%	AADDD	3.7%	ADADDD<br>DAADDD	1.9%	DDAD
			3.8%	AADD	2.7%	ADAADD<br>ADADAD<br>DAAADD<br>DAADAD	1.8%	DAAD<br>DADA
			2.3%	ADADD	2.7%	AADADD<br>ADAADD<br>DAAADD	1.6%	DDDD
			1.6%	AADADD	2.5%	ADADD<br>ADDAD		
			1.6%	ADADDD<br>DAADDD	2.4%	ADDADD<br>DADADD		
			1.6%	DAAADD	2.4%	ADADDD<br>ADDADD<br>ADDDAD		
			1.0%	DDAD	2.1%	AADD		
					1.8%	DAD		
					1.7%	DAAD<br>DADA		
					1.7%	ADAD<br>DAAD		
					1.5%	ADDDAD<br>DADDAD		
					1.5%	ADAADD<br>ADADAD<br>ADDAAD		
					1.5%	DAAADD<br>DAADAD<br>DADAAD		
					1.1%	ADDDD		

D: GlcN; A: GlcNAc.

Supplementary Table 5│Relative abundancies (molar fraction) of patterns at the reducing and non-reducing ends of oligomers in the early phase of hydrolysis (15 min). Oligomers were produced from chitosans with F_A 0.11-0.50 using CSN174, CSN-7M, CSN-MN, and CSN-MHKI. Shown are the mean values with standard deviations of three independent measurements of three independent enzyme batches.

	**reducing ends unknown pattern**	**reducing end dyad frequencies**			
		**AA**	**AD**	**DA**	**DD**
**CSN-174 F_A 0.11**	0.0 ± 0.0%	0.1 ± 0.1%	1.1 ± 0.1%	9.8 ± 6.2%	89.0 ± 6.3%
**CSN-174 F_A 0.50**	0.0 ± 0.0%	1.1 ± 1.3%	4.4 ± 1.1%	37.4 ± 6.4%	57.1 ± 6.4%
**CSN-7M F_A 0.11**	0.5 ± 0.5%	0.5 ± 0.6%	2.1 ± 0.4%	1.8 ± 0.6%	95.5 ± 0.9%
**CSN-7M F_A 0.50**	0.7 ± 0.9%	0.4 ± 0.2%	4.7 ± 1.6%	1.7 ± 0.5%	93.2 ± 1.7%
**CSN-MN F_A 0.11**	0.0 ± 0.0%	0.7 ± 1.0%	1.4 ± 1.5%	0.9 ± 0.9%	97.0 ± 2.3%
**CSN-MN F_A 0.50**	0.7 ± 1.0%	0.6 ± 0.9%	3.8 ± 5.3%	2.3 ± 3.2%	93.3 ± 9.4%
**CSN-MHKI F_A 0.11**	0.0 ± 0.0%	0.1 ± 0.1%	0.7 ± 0.4%	9.0 ± 12.0%	90.2 ± 12.4%
**CSN-MHKI F_A 0.50**	8.5 ± 11.2%	4.1 ± 4.2%	5.9 ± 5.5%	6.0 ± 4.3%	83.9 ± 13.8%
	**non-reducing ends unknown pattern**	**non-reducing end dyad frequencies**			
		**AA**	**AD**	**DA**	**DD**
**CSN-174 F_A 0.11**	0.0 ± 0.0%	0.0 ± 0.1%	1.4 ± 0.9%	6.0 ± 1.8%	92.6 ± 2.4%
**CSN-174 F_A 0.50**	0.0 ± 0.0%	1.5 ± 1.1%	4.1 ± 1.2%	12.7 ± 4.9%	81.7 ± 4.5%
**CSN-7M F_A 0.11**	0.4 ± 0.5%	0.3 ± 0.3%	1.4 ± 0.1%	6.9 ± 1.1%	91.4 ± 1.2%
**CSN-7M F_A 0.50**	0.0 ± 0.0%	0.6 ± 0.3%	3.0 ± 1.0%	21.5 ± 2.9%	74.9 ± 3.8%
**CSN-MN F_A 0.11**	0.2 ± 0.2%	0.7 ± 1.0%	0.4 ± 0.3%	2.7 ± 3.6%	96.2 ± 3.5%
**CSN-MN F_A 0.50**	0.0 ± 0.0%	0.9 ± 1.3%	2.6 ± 3.7%	9.6 ± 13.6%	86.6 ± 18.6%
**CSN-MHKI F_A 0.11**	0.0 ± 0.0%	0.0 ± 0.1%	1.4 ± 0.5%	6.8 ± 1.1%	91.9 ± 1.6%
**CSN-MHKI F_A 0.50**	8.1 ± 11.5%	4.1 ± 4.2%	5.3 ± 3.0%	14.4 ± 9.3%	76.3 ± 15.5%

D: GlcN; A: GlcNAc.

Supplementary Table 6 | Pattern distribution of mono-acetylated oligomers with DP 3-6 produced with CSN-7M. Given are the molar fractions of each oligomer at the end point of hydrolysis. Shown are the mean values with standard deviations of three independent measurements of three independent enzyme batches.

oligomer	pattern of acetylation	mole fraction			
		F_A 0.11	F_A 0.19	F_A 0.35	F_A 0.50
**D_4**	DDDD	0.4 ± 0.0%	0.5 ± 0.0%	1.2 ± 0.8%	0.8 ± 0.2%
**D_2A_1**	DDA	0.4 ± 0.0%	0.4 ± 0.0%	0.3 ± 0.0%	0.4 ± 0.2%
	DAD	0.4 ± 0.0%	0.4 ± 0.0%	0.2 ± 0.0%	0.6 ± 0.5%
	ADD	3.0 ± 0.1%	3.7 ± 0.2%	1.5 ± 0.1%	4.5 ± 2.7%
**D_3A_1**	DDDA	0.3 ± 0.0%	0.3 ± 0.0%	0.4 ± 0.0%	0.3 ± 0.2%
	DDAD	1.2 ± 0.2%	1.3 ± 0.3%	0.3 ± 0.2%	0.4 ± 0.0%
	DADD	2.9 ± 0.3%	4.1 ± 0.6%	3.8 ± 1.1%	5.2 ± 1.3%
	ADDD	6.3 ± 0.2%	8.7 ± 0.3%	11.3 ± 1.1%	11.2 ± 2.4%
**D_4A_1**	DDDDA	0.0 ± 0.0%	0.1 ± 0.1%	0.6 ± 0.1%	0.0 ± 0.1%
	DDDAD	0.0 ± 0.0%	0.0 ± 0.0%	0.0 ± 0.0%	0.3 ± 0.5%
	DDADD	1.8 ± 0.1%	3.5 ± 0.3%	6.5 ± 0.6%	3.4 ± 1.8%
	DADDD	5.8 ± 0.5%	7.4 ± 0.3%	20.2 ± 0.7%	13.7 ± 7.5%
	ADDDD	0.1 ± 0.1%	0.2 ± 0.2%	1.3 ± 1.3%	0.2 ± 0.1%
**D_5A_1**	DDDDDA	0.0 ± 0.0%	0.0 ± 0.0%	0.1 ± 0.1%	0.0 ± 0.0%
	DDDDAD	0.0 ± 0.0%	0.0 ± 0.0%	0.0 ± 0.1%	0.0 ± 0.0%
	DDDADD	0.0 ± 0.0%	0.0 ± 0.0%	0.0 ± 0.0%	0.2 ± 0.3%
	DDADDD	0.0 ± 0.0%	0.1 ± 0.2%	3.6 ± 0.6%	1.7 ± 1.2%
	DADDDD	0.0 ± 0.0%	0.0 ± 0.0%	0.1 ± 0.1%	0.1 ± 0.1%
	ADDDDD	0.0 ± 0.0%	0.0 ± 0.1%	0.4 ± 0.4%	0.1 ± 0.1%

D: GlcN; A: GlcNAc.

Supplementary Table 7 | MS parameters for tandem MS of re-*N*-acetylated ^{18}O-labelled chitosan oligomers. Shown is an exemplary setup, as the elution time was adjusted for each set of measurement.

Elution time [min]	Target mass [m/z]	Mass [m/z]	Sequence	Width	Cut-Off	Amplitude [%]
0-4	200	-	-	-	-	-
4-7.5	420	430.2	A1D1	2	116	85
7.5-11.5	630	633.2	A2D1	2	171	90
7.5-11.5	630	636.2	A1D2	2	172	90
11.5-15.5	840	836.3	A3D1	1.5	226	100
11.5-15.5	840	839.3	A2D2	1.5	227	100
11.5-15.5	840	842.3	A1D3	1.5	227	100
15.5-19	1040	1039.4	A4D1	1.5	281	100
15.5-19	1040	1042.4	A3D2	1.5	281	100
15.5-19	1040	1045.4	A2D3	1.5	282	100
15.5-19	1040	1048.4	A1D4	1.5	283	100
19-23.5	1240	1242.5	A5D1	1.5	335	110
19-23.5	1240	1245.5	A4D2	1.5	336	110
19-23.5	1240	1248.5	A3D3	1.5	337	110
19-23.5	1240	1251.5	A2D4	1.5	338	110
19-23.5	1240	1254.5	A1D5	1.5	339	110

D: GlcN; A: GlcNAc.

Supplementary Table 8 | List of pectin samples used.

**Pectin substrate**	**Abbreviation**	**Supplier**	**DM**
polymeric pectin C	PC	Carl Roth GmbH (Karlsruhe, Germany)	≈ 70%
polymeric pectin from apple	PAP	Carl Roth GmbH (Karlsruhe, Germany)	≈ 38%
polymeric pectin from citrus peel	PCP	Fluka (Buchs, Switzerland)	63-66%

DM: degree of methyl esterification.

Supplementary Table 9 | List of pectin degrading enzymes used.

**Enzyme**	**Abbreviation**	**Supplier**
endo-Polygalacturonanase from *Aspergillus niger*	PG	Megazyme (Bray, Ireland) Lot No. MPG00901
Pectate lyase from *Aspergillus sp*.	PL	Megazyme (Bray, Ireland) Lot No 10801

Supplementary Table 10 | HILIC parameters for LC- MS measurements of pectin oligomers.

**time [min]**	**eluent**
0-2.5	100% A
2.5-15	linear gradient to 100% B
15-17	100% B
17-17.5	linear gradient to 100% A
17.5-20	100% A
